# A systematic transcriptomic atlas and predictive framework for metabolite-driven T cell state transitions

**DOI:** 10.1016/j.isci.2026.117527

**Published:** 2026-09-18

**Authors:** Yiyang Xu, Yiheng Zhao, Sizhe Yang, Huimin Li, Xiangrong Diao, Qianqian Zhang, Shuting Yu, Shuai Wang, Fangying Jiang, Qiang Wang, Yuhua Ma, Bo Jiao, Yun Tan, Yabin Liu

**Affiliations:** 1 Shanghai Institute of Hematology, State Key Laboratory of Medical Genomics, National Research Center for Translational Medicine at Shanghai, Ruijin Hospital Affiliated to Shanghai Jiao Tong University School of Medicine, Shanghai, China; 2 College of Health Science and Technology, Shanghai Jiao Tong University School of Medicine, Shanghai, China; 3 Shu Chien-Gene Lay Department of Bioengineering, Jacobs School of Engineering, University of California, San Diego, La Jolla, CA, USA; 4 Department of Nephrology, Kunshan Hospital Affiliated to Nanjing University of Chinese Medicine, Kunshan, Jiangsu, China

**Keywords:** T cell, metabolites, DRUG-seq, perturbation atlas, predictive framework

## Abstract

Gut microbial and endogenous metabolites are key regulators at the interface of host metabolism and immunity, yet their global effects on human T cell states remain poorly defined. Here, we profile primary human T cell responses to 364 endogenous and microbiota-derived metabolites using high-throughput digital RNA with perturbation of genes sequencing (DRUG-seq), revealing structured transcriptional trajectories spanning baseline, metabolically primed, and highly activated states. We develop DRUG-seq-PerturbFormer, a multi-task deep learning framework that quantitatively captures perturbation magnitude and directionality across these states. This analysis identifies a subset of metabolites that robustly reprogram T cell transcriptional programs. In a dextran sulfate sodium (DSS)-induced colitis model, representative candidates attenuated disease severity and were associated with suppression of inflammatory programs and partial restoration of immune homeostasis. Collectively, these findings show that metabolites act as signals shaping T cell function via transcription, nominating immunomodulatory targets for inflammatory diseases.

## Highlights

DRUG-seq maps human T cell transcriptional responses to 364 metabolites
• DRUG-seq-PerturbFormer robustly predicts and ranks metabolite perturbation strength
• Metabolites reprogram T cells along a structured activation continuum
• Prioritized metabolites alleviate acute colitis and restore immune homeostasis

## Graphical abstract

**Figure:**
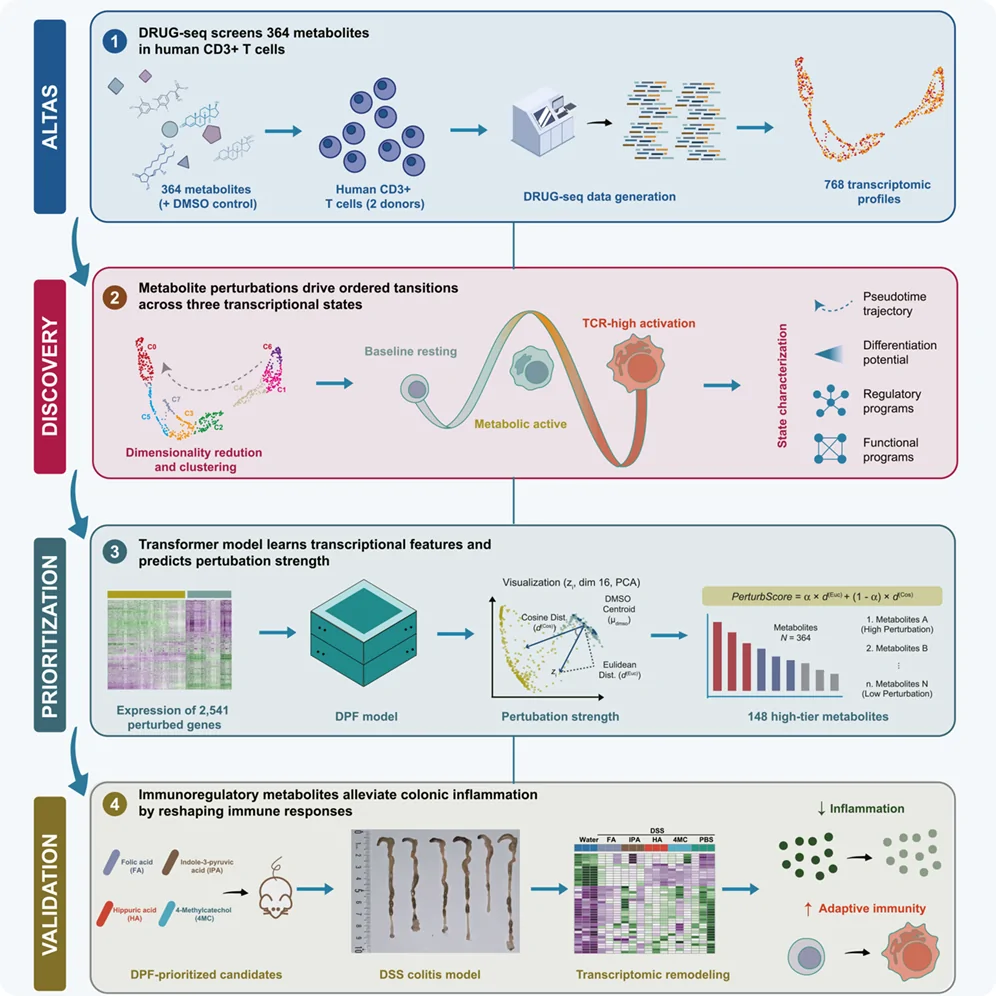

## Teaser

Immunology; Bioinformatics; Transcriptomics

## Introduction

The gut microbiota exerts a broad influence on host physiology by shaping metabolic, immunological, and inflammatory processes through a chemically diverse repertoire of small molecules.^1^ Many of these compounds arise from microbial transformation of dietary substrates, whereas others are synthesized endogenously or generated through host-microbe co-metabolism.^2^^,^^3^ These metabolites form a complex signaling interface that mediates bidirectional communication between microbial communities and the immune system.

T cells are central coordinators of adaptive immunity, and their functional states are stringently regulated by the metabolic environments they inhabit.^4^ Beyond antigen recognition through the T cell receptor (TCR), nutrient availability and metabolite sensing act as additional regulatory inputs that influence T cell activation and differentiation.^5^^,^^6^ The gastrointestinal tract represents a primary interface where T cells interact with a diverse chemical milieu of endogenous and microbiota-derived metabolites.^7^ Accumulating studies demonstrate that such metabolites modulate multiple aspects of T cell biology, including activation, effector differentiation, cytokine production, and tissue homeostasis.^8^ For instance, short-chain fatty acids (SCFAs) from gut microbiota, including acetate, propionate, and butyrate, promote colonic regulatory T (Treg) cell differentiation through GPR43 signaling and histone deacetylase inhibition.^9^ SCFAs also modulate effector T cell subsets and attenuate airway inflammation by influencing immunometabolism.^10^ Tryptophan-derived indole metabolites similarly engage the aryl hydrocarbon receptor to regulate T helper 17 (Th17)/Treg balance and maintain mucosal homeostasis.^11^ Secondary bile acids generated from microbial modification of host bile acids signal through FXR and TGR5 to modulate epithelial integrity and innate immunity.^12^^,^^13^ Beyond classical metabolite classes, metabolomic studies have linked host-microbe co-metabolites, such as hippurate, to systemic inflammation and metabolic status,^14^ and polyamine metabolism has been implicated in T cell exhaustion and response to CAR-T (chimeric antigen receptor T-cell immunotherapy) therapy.^15^^,^^16^ Despite these advances, the overarching organizational principles that govern how diverse metabolites reshape T cell transcriptional states at scale, as well as how such information can be systematically leveraged for translational prioritization, remain incompletely defined.

Technical limitations have also contributed to this gap. Although recent advances in high-throughput perturbation profiling have enabled large-scale mapping of ligand-induced transcriptional responses in immune cells,^17^^,^^18^ these approaches have rarely been applied to microbial metabolites. Existing studies typically assess small numbers of metabolites, rely on bulk readouts that obscure heterogeneity, or lack standardized analytical frameworks for broad comparison across conditions. Notably, no unified resource currently exists that systematically compiles immune-related transcriptional signatures elicited by gut microbial metabolites. This absence of an integrated dataset limits the ability to identify convergent or divergent transcriptional programs, hinders cross-study comparability, and constrains efforts to quantitatively prioritize metabolite candidates. In parallel, computational models capable of predicting transcriptional outcomes induced by untested metabolites are still in their infancy. Current machine-learning approaches are either trained on limited perturbation datasets or insufficiently capture the complexity of immune regulatory networks, making it challenging to extrapolate beyond experimentally assayed compounds or to infer latent determinants of metabolite-driven T cell state transitions.

To address these challenges and systematically map the metabolic-transcriptional landscape of T cells, we utilized digital RNA with perturbation of genes sequencing (DRUG-seq), a high-throughput technology developed to sequence total RNA directly from cell lysates in a 384-well format, bypassing cost-prohibitive purification steps.^19^^,^^20^^,^^21^ This allowed us to profile the responses of human T cells to a library of 364 metabolites. This large-scale screen revealed that metabolite-induced transcriptional responses are not randomly distributed, but instead organized into a limited number of recurring transcriptional programs, prominently involving coordinated activation of metabolic pathways and TCR-associated signaling modules.

In parallel with advances in high-throughput perturbation profiling, deep learning-based modeling has emerged as a powerful approach for predicting complex transcriptional responses from high-dimensional gene expression data. In particular, neural network architectures based on multi-task transformers have demonstrated a strong capacity to capture gene-gene dependencies and generalize to unseen perturbations.^22^ In this context, we developed DRUG-seq-PerturbFormer (DPF), a deep learning framework that integrates experimental perturbation data with predictive modeling, to enable quantitative prioritization of metabolite-induced transcriptional effects across T cell states.

Together, this integrated experimental and computational framework generates a comprehensive, high-resolution atlas of metabolite-induced transcriptional responses, providing a foundation for exploring how diverse metabolites reshape human T cell programs and for guiding downstream functional and translational studies.

## Results

### Systematic perturbation profiling reveals T cell transcriptional programs induced by 364 metabolites

To systematically characterize how endogenous and microbiota-associated metabolites modulate T cell transcriptional states, we established a high-throughput perturbation profiling platform integrating DRUG-seq, transcriptional-state characterization, differential expression analysis, and downstream modeling (Figures 1A and S1 and Table S1). Primary human activated CD3^+^ T cells were treated with 364 endogenous or gut microbiota-derived metabolites, alongside DMSO controls. Transcriptomic changes were quantified by DRUG-seq in biological duplicates (Figure S2A), generating a large-scale perturbation dataset for subsequent integrative analyses.

**Figure 1:**
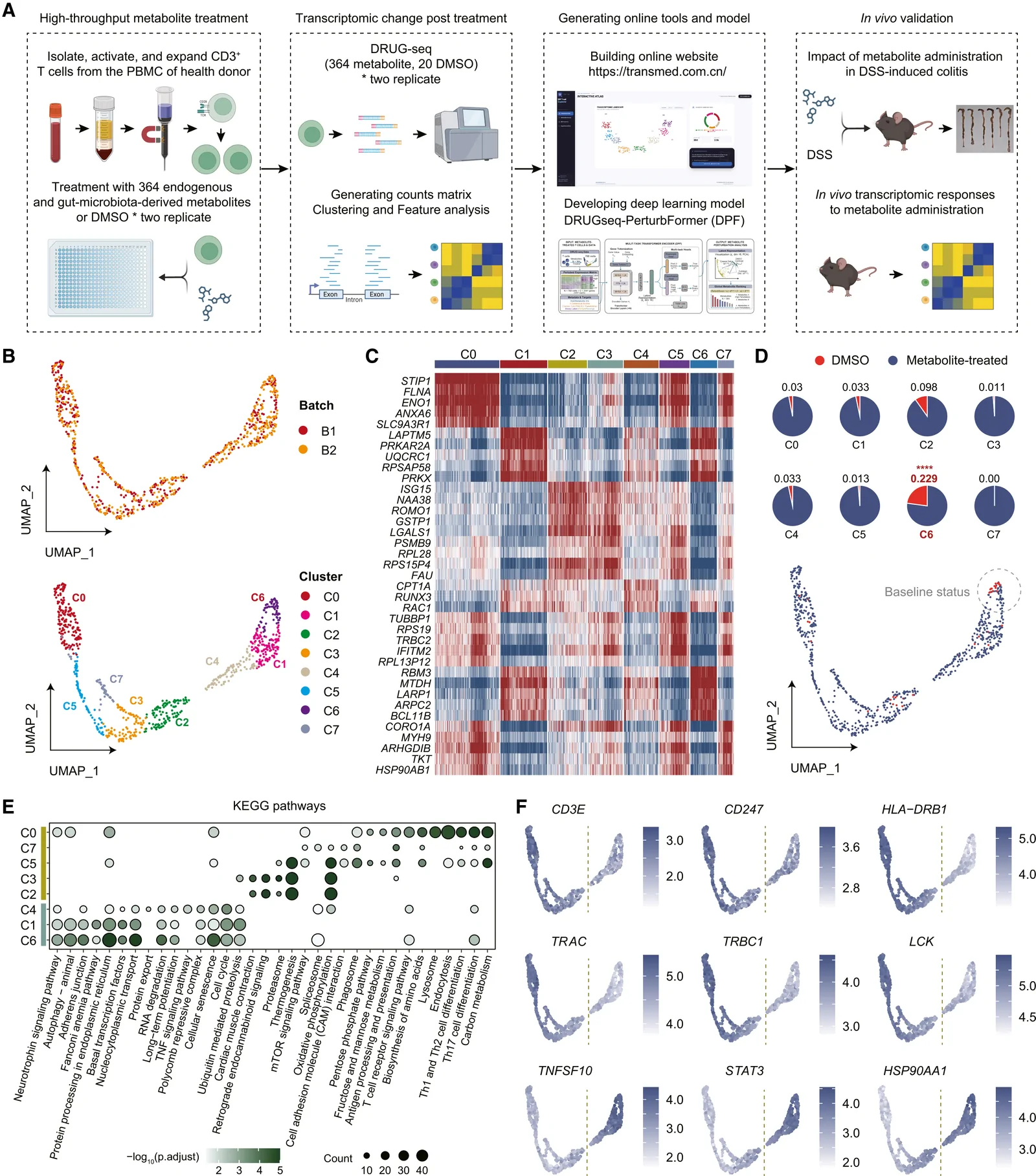
Systematic transcriptomic perturbation profiling of 364 endogenous and gut microbial metabolites reveals distinct T cell regulatory modules (A) Schematic of the experimental design for high-throughput metabolite treatment. Peripheral blood mononuclear cells (PBMCs) from healthy donors were isolated, activated, and expanded. Cells were treated with 364 endogenous and gut microbiota-derived metabolites or DMSO (two replicates) and subjected to DRUG-seq analysis. This process generated a counts matrix, followed by clustering and feature analysis. Perturbation signatures were integrated into an online resource (https://www.transmed.sh.cn) and used to train a deep learning model (DRUG-seq-PerturbFormer, DPF). Key metabolites were further assessed in a DSS-induced colitis model. (B) UMAP clustering of the T cell transcriptomes based on batch (D1 and D2) and cluster (C0–C7) distribution, showing distinct patterns of transcriptional responses to metabolite treatments. (C) Heatmap depicting differential gene expression across clusters (C0–C7), with several genes highlighted that show strong differential expression in specific clusters. (D) Dot plots showing the differential gene expression in response to DMSO (control) or metabolite treatment. Fisher’s exact test was used to evaluate the association between transcriptional clusters and treatment groups. Statistical significance is indicated by asterisks (∗∗*p* < 0.0001). (E) KEGG pathway enrichment analysis reveals the top-enriched pathways across the clusters. Enrichment significance was assessed using a hypergeometric test, with *p* values adjusted for multiple comparisons using the Benjamini-Hochberg (BH) method. (F) Expression of key genes within clusters, including *CD3E*, *CD247*, *HLA-DRB1*, *TRAC*, *TNFSF10*, *STAT3*, *LCK*, and *HSP90AA1*, as visualized through UMAP plots. Gene expression is represented on a color scale indicating the normalized expression levels.

Dimensionality reduction by uniform manifold approximation and projection (UMAP) revealed a continuous yet structured landscape of metabolite-induced transcriptional variation (Figure 1B and Table S2). Unsupervised clustering identified eight discrete transcriptional states (C0–C7), representing distinct response modules elicited by metabolite perturbation (Figure 1B, bottom). These clusters captured coordinated expression programs spanning activation, cytokine signaling, metabolic adaptation, and stress responses.

Marker gene analysis highlighted unique transcriptional signatures characterizing each state (Figure 1C). Among these states, C0 showed features of a strongly activated program, whereas C6 was characterized by prominent interferon- and stress-associated signatures. Cluster composition analysis further showed that DMSO controls were relatively enriched in C6, whereas metabolite-treated cells predominated across most other clusters (Figures 1D and S2B). These findings indicate that a substantial fraction of metabolites elicit measurable and state-specific transcriptional remodeling in T cells.

To further interpret the biological relevance of each cluster, we conducted Kyoto Encyclopedia of Genes and Genomes (KEGG) enrichment analysis on cluster-defining genes (Figure 1E and Table S3). The resulting landscape revealed heterogeneous pathway engagement. For instance, cluster C0, which displayed a strongly activated transcriptional phenotype, was enriched for pathways involved in TCR signaling, antigen processing and presentation, cytokine-cytokine receptor interaction, and Th1/Th2/Th17 differentiation, indicating coordinated activation of T cell effector programs. By contrast, C1 and C6 were enriched for interferon- and stress-associated processes but represented distinct transcriptional clusters. C6 contained most DMSO-treated samples and was therefore designated as the baseline cluster. Although C1 and C4 were not similarly enriched for DMSO, the metabolites in these clusters exhibited transcriptional perturbation patterns similar to those observed in C6. Inspection of individual genes further demonstrated the diversity of T cell states induced by metabolite perturbation (Figure 1F). Key surface markers and signaling molecules, including *CD3E*, *CD247*, *TRAC*, *TRBC1*, *LCK*, *STAT3*, and immune regulators such as *HLA*-*DRB1*, *TNFSF10*, and *HSP90AA1*, showed distinct expression gradients across the UMAP space, aligning with the inferred transcriptional modules.

### Metabolite perturbations converge on three higher order T cell activation states

To dissect how individual metabolites modulate T cell transcriptional programs, we first examined differential gene expression signatures across all treatments. The cluster-specific transcriptional and pathway programs converged into three higher order response states, termed baseline resting, metabolic active, and TCR-high activation (Table S4). Across metabolites, the numbers of upregulated and downregulated genes were strongly correlated (Pearson’s *r* = 0.98, *p* < 2.2 × 10^−16^), with transcriptional perturbation progressively increasing from the baseline resting state to the metabolic active and TCR-high activation states (Figure 2A). Metabolites were then ranked within each state according to total differentially expressed gene (DEG) count, revealing marked variation in perturbation strength among metabolites occupying the same transcriptional state (Figure 2B). Within the TCR-high activation state, top-ranked metabolites such as 2-methylbutanoic acid, tocotrienol, resveratrol, and indole-3-pyruvic acid were associated with strong induction of activation-related genes, including *IL2RA*, *CD5*, *LCK*, and *CCR7* (Figures 2C and S3A). By contrast, metabolites assigned to the metabolic active state preferentially enhanced bioenergetic genes, such as *NDUFA11* and *NDUFB8*, consistent with selective engagement of metabolic programs. Together, these data suggest that chemically diverse metabolites can be organized into a limited number of recurrent functional response states.

**Figure 2:**
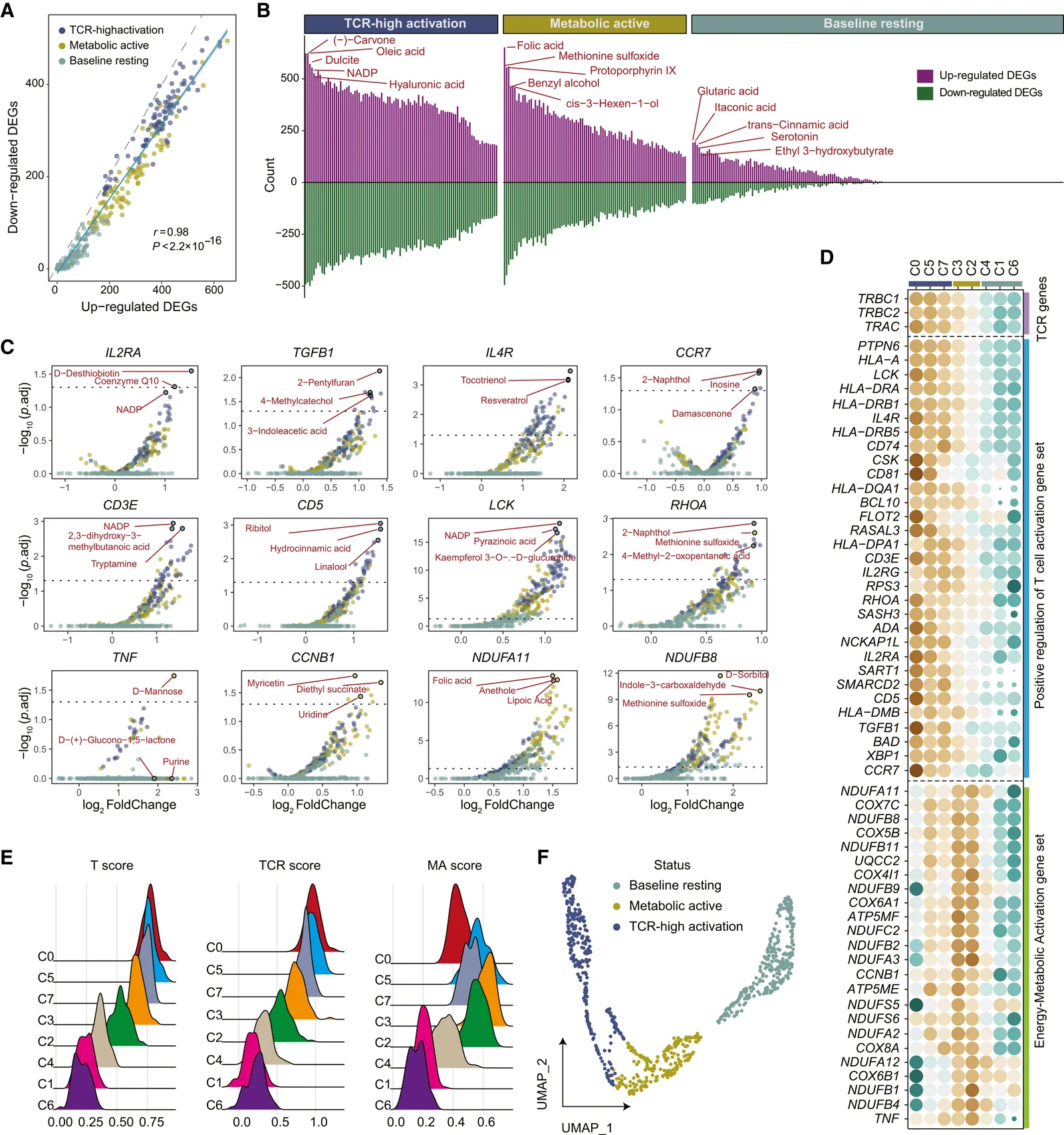
Metabolite-induced T cell activation and transcriptomic remodeling (A) Relationship between the numbers of upregulated and downregulated differentially expressed genes (DEGs) induced by each metabolite relative to DMSO. Points are colored by transcriptional state; the solid line shows the linear fit with 95% confidence interval, and the dashed line indicates equal DEG numbers. Pearson’s *r* and two-sided *p* value are shown. (B) Bar plot summarizing, for each of the 364 metabolites, the number of DEGs compared with DMSO controls. Bars are stacked by direction of change, with purple indicating upregulated genes and green indicating downregulated genes. Compounds are ordered by total DEG count and grouped into three transcriptional response states. (C) Volcano plots showing the differential expression of genes in response to various metabolites. Each plot represents the log_2_ (fold change) versus the significance (adjusted *p* value) for different metabolites. Notable metabolites are highlighted in red, with the corresponding gene names marked for clarity. *p* values were calculated using the DESeq2 Wald test and adjusted for multiple comparisons using the BH method. The dashed horizontal line indicates an adjusted *p* value of 0.05. Representative metabolites are highlighted and labeled in red. (D) Heatmap of gene expression for genes involved in T cell activation and metabolic processes. Rows represent selected genes from the T cell receptor (TCR) and positive regulation of T cell activation gene sets. Columns represent the status of different clusters, categorized into baseline resting, metabolically active, and TCR-high activation. (E) Distribution of T cell scores (T score), TCR scores (TCR score), and metabolic activation (MA) scores across the clusters. (F) UMAP projection showing the status of T cells based on their baseline resting, metabolically active, and TCR-high activation states. Different colors represent cells in distinct metabolic and activation states, illustrating the shift in T cell subsets in response to metabolite treatment.

To define the gene programs underlying these higher order states, we merged significantly DEGs from all pairwise comparisons to construct a perturbation-responsive gene universe (*N* = 2,541) (Figure S3B). KEGG enrichment analysis of this gene universe revealed that upregulated genes were significantly enriched in T cell activation pathways (e.g., TCR signaling and Th1/Th2 differentiation), while downregulated genes were associated with more basal cellular and metabolic processes (Figure S3C). When mapped back onto the eight transcriptional clusters identified in Figure 1, these genes showed clear state-specific organization: activation-associated genes were most prominently expressed in cluster C0, whereas metabolic genes mapped preferentially to clusters C2 and C3 (Figure 2D). This result links the three-state framework to the finer cluster structure defined in the initial perturbation screen.

To further quantify these states, we computed three signature-based metrics, including a T cell activation score (T score), TCR signaling score (TCR score), and metabolic activation (MA) score (Figures 2E and S3D–S3F). Cluster C0 showed the highest T score and TCR score, whereas clusters C2 and C3 displayed elevated MA scores, highlighting functionally distinct metabolite-driven phenotypes. When projected back onto the transcriptional landscape, these scores organized cells into the three major states—baseline resting, metabolic active, and TCR-high activation, indicating that metabolite perturbations can be interpreted as shifts along a structured activation continuum rather than as unrelated transcriptional outcomes (Figure 2F).

### Hierarchical regulatory programs organize metabolite-induced T cell state transitions

To further define the regulatory logic and functional consequences underlying the three metabolite-induced states, we integrated regulatory network inference, cell identity enrichment, and trajectory modeling. Regulon-level analysis revealed a structured hierarchy of transcriptional regulators across the state continuum. The baseline resting state was marked by higher activity of homeostasis-associated regulators, including *STAT3* and *PURA*, whereas the intermediate metabolic active state showed preferential enrichment of *KLF2*- and *MXD3*-associated regulons, consistent with a metabolically primed intermediate program. In contrast, the TCR-high activation state was associated with increased activity of stress- and inflammation-related regulators, including *ATF4*, *SREBF2*, *RELA*, and *JUND* (Figures 3A and 3B). Together, these data suggest that the three higher order states are organized by a progressive regulatory hierarchy rather than by unrelated transcriptional signatures.

**Figure 3:**
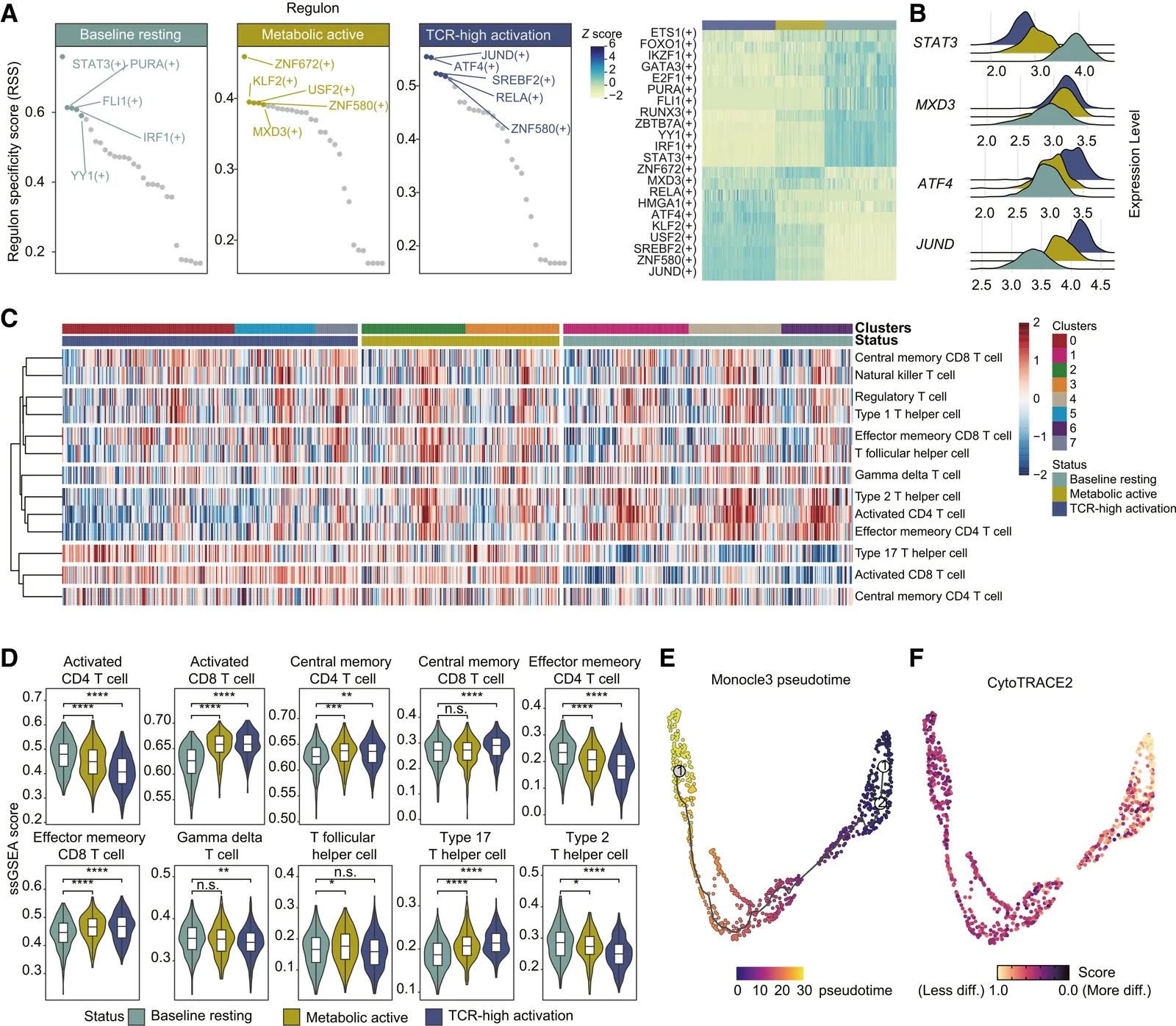
Transcriptional regulatory landscapes and functional subset dynamics across metabolite-induced T cell states (A) Regularity score for key transcription factors in different T cell states. These transcription factors are shown to display specific regularity across the T cell subsets, as measured by *Z* scores. (B) Gene expression analysis for *STAT3*, *MXD3*, *ATF4*, and *JUND*, key transcription factors associated with T cell activation and differentiation. (C) Heatmap of single-sample gene set enrichment analysis (ssGSEA) scores, quantifying the enrichment of specific T cell subtype signatures across the identified transcriptional clusters. (D) Violin plots showing the relative enrichment of T cell subsets distribution based on gene set variation analysis (GSVA) scores for various T cell types. The width of the violin plots represents the kernel density estimation of the data distribution. Within each violin, the box extends from the 25th percentile (Q1) to the 75th percentile (Q3), with the central line indicating the median. The whiskers extend to the minimum and maximum values within 1.5 × IQR (interquartile range) from the lower and upper quartiles, respectively. (E) Pseudotime trajectory of T cell differentiation based on Monocle3 analysis, indicating the progressive states of T cell differentiation under different conditions, from baseline resting to metabolically active and TCR-high activation states. (F) CytoTRACE2-based pseudotime analysis showing the differentiation trajectories of T cells in response to metabolite treatment. Cells are color-coded based on differentiation state, with scores indicating the degree of differentiation. Lower scores represent less differentiation, while higher scores indicate more differentiated states. Statistical significance was assessed using a two-sided Wilcoxon rank-sum test (∗*p* < 0.05, ∗∗*p* < 0.01, ∗∗∗*p* < 0.001, and ∗∗∗∗*p* < 0.0001).

Consistent with this regulatory organization, gene set variation analysis revealed state-dependent remodeling of T cell functional identity. We first quantified the enrichment of 13 T cell subtype signatures across the transcriptional landscape (Figure 3C). When these signatures were compared across the three major states, distinct patterns emerged between CD4^+^ and CD8^+^ compartments (Figure 3D). Activated CD8^+^ T cell and effector memory CD8^+^ T cell signatures increased stepwise from baseline resting to TCR-high activation (*p* < 0.0001), whereas activated CD4^+^ and effector memory CD4^+^ T cell signatures declined progressively across the same transition (*p* < 0.0001). These data indicate that metabolite perturbations differentially reshape CD4^+^ and CD8^+^ programs rather than uniformly amplifying all activated T cell states. Meanwhile, both CD4^+^ and CD8^+^ central memory signatures showed an upward trend across states, suggesting partial preservation or enrichment of memory-associated programs. Among helper T cell signatures, Th1 remained relatively stable, whereas Th17 increased progressively, pointing to selective remodeling of helper-lineage programs along the activation continuum.

To determine whether these relationships reflected a continuous progression rather than discrete and unrelated cell groups, we next reconstructed the state trajectory using two independent analytical frameworks. Both approaches supported a continuous path originating from the baseline-enriched region, traversing an intermediate bridge dominated by metabolically active states, and terminating in the highly activated branch (Figures 3E, 3F, and S4A–S4C). In parallel, differentiation-potential analysis indicated a progressive reduction in transcriptional stemness from resting-like to highly activated states. Notably, metabolites with weaker perturbation strength remained enriched near the trajectory root, whereas stronger perturbagens were associated with cells positioned toward the terminal branch. Together, these analyses support a model in which metabolite-induced T cell states are arranged along a progressive activation continuum governed by hierarchical regulatory programs.

### A multi-task transformer model learns, ranks, and generalizes metabolite perturbation strength

Having defined the major metabolite-responsive T cell states and their associated activation continuum, we next asked whether these perturbational features could be learned in a predictive framework. To quantitatively model the effects of endogenous and gut microbial metabolites on T cell transcriptional states, we developed a multi-task transformer encoder, DPF, which integrates gene-level expression profiles with multi-output prediction heads (Figure 4A). To capture both perturbation strength and state progression, we selected three continuous metrics—T cell activation score (T score), differentiation potential (CytoTRACE2), and pseudotime—as regression targets, as these scores collectively reflect the depth of T cell activation and developmental progression. Furthermore, to sharpen the contrast during model training, we merged the metabolic active and TCR-high activation states into a unified perturbed label to compare against the resting state. This multi-task strategy enables a unified latent representation of metabolite-induced transcriptional changes. Training trajectories showed stable optimization of both regression and classification losses, with convergence achieved around epoch 47 (Figure 4B). Model performance was further supported by strong agreement between predicted and observed values for all three continuous metrics, with Pearson’s *r* = 0.96 for T score, 0.98 for CytoTRACE2, and 0.94 for pseudotime (all *p* < 2.2 × 10^−16^), together with a high area under the curve (AUC) of 0.987 (95% confidence interval [CI] 0.976–0.998) for distinguishing perturbed from resting conditions (Figures S5A–S5D).

**Figure 4:**
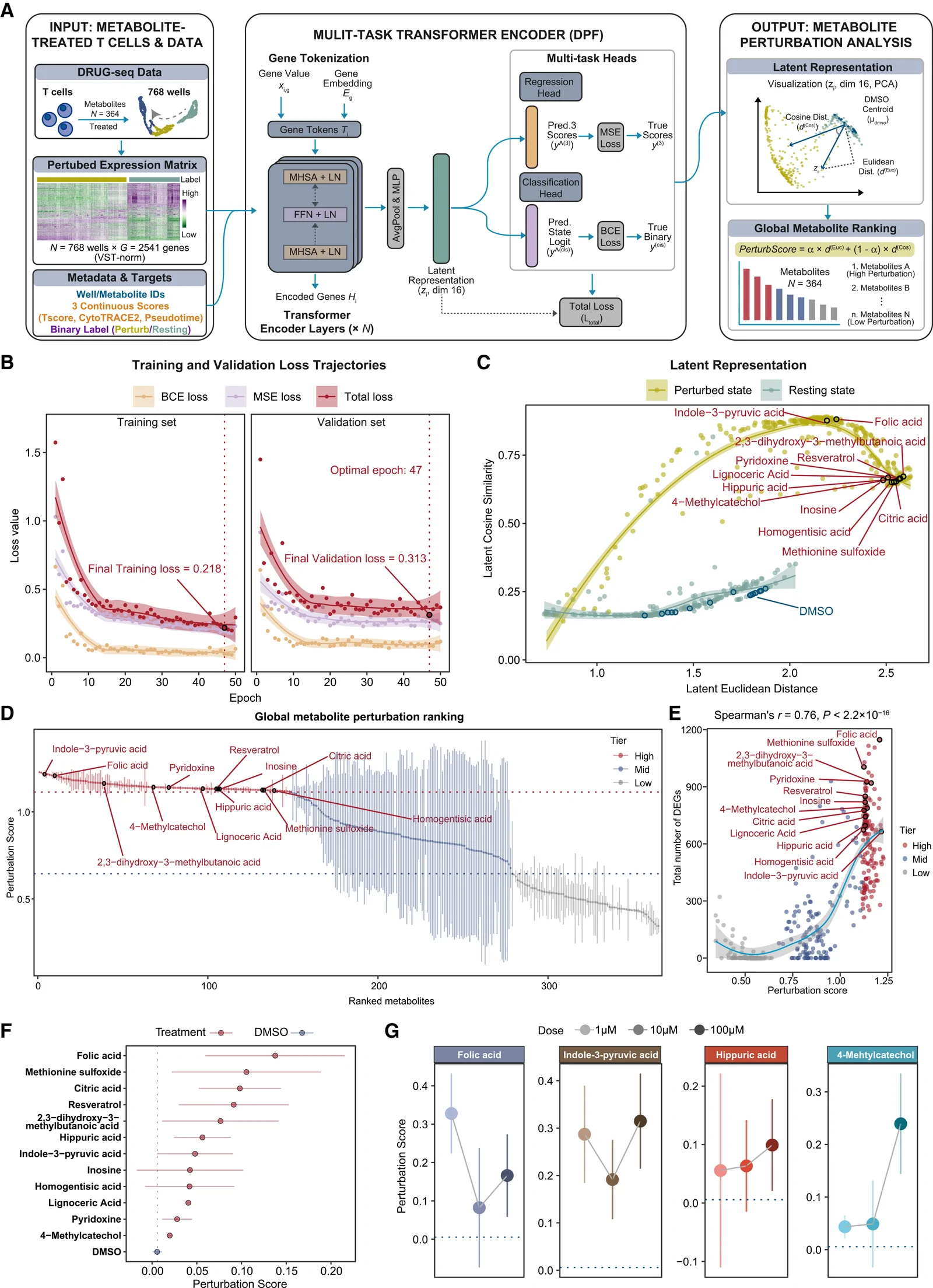
Development and validation of the DRUG-seq-PerturbFormer model for metabolite perturbation analysis (A) Overview of the pipeline using the DRUG-seq-PerturbFormer (DPF). The input includes metabolite-treated T cell data, consisting of 768 wells and 2,541 genes. The data undergoes gene tokenization and encoding, followed by multi-task analysis with both regression and classification heads. The model performs multi-task analysis to predict transcriptional outcomes and generate global metabolite rankings. (B) Training and validation loss trajectories for the DPF model, showing the convergence of both binary cross-entropy (BCE) loss and mean squared error (MSE) loss over training epochs. (C) Latent representation of metabolite perturbations visualized using Euclidean and cosine distance metrics, comparing the resting state and perturbed state of treated T cells. (D) Global ranking of metabolites based on perturbation scores, highlighting metabolites with the highest and lowest impact on T cell transcriptional reprogramming. The perturbation score indicates the magnitude of each metabolite’s effect on the T cell transcriptome, with metabolites clustered based on their relative potency. (E) Relationship between DPF perturbation score and total DEG count across metabolites. Each point represents one metabolite and is colored according to the DPF-defined perturbation tier. The fitted trend line is shown with its 95% confidence interval. Selected high-ranking metabolites are labeled. Spearman’s correlation coefficient and two-sided *p* value are shown. (F) Validation of model generalization using independent bulk RNA-seq data. DPF-predicted global perturbation scores for T cells treated with 12 representative metabolites identified as active perturbagens (e.g., indole-3-pyruvic acid, folic acid, and 4-methylcatechol). The model consistently assigns significantly higher perturbation scores to these metabolite-treated samples compared to the DMSO baseline, confirming its robustness in recognizing activation signatures across different data modalities. Data are represented as mean ± standard deviation (SD). (G) Dose-dependent prediction sensitivity. Trajectories of predicted perturbation scores for four high-confidence candidate metabolites (folic acid, indole-3-pyruvic acid, hippuric acid, and 4-methylcatechol) across increasing concentrations (1, 10, and 100 μM). The model captures the progressive escalation of T cell perturbation intensity corresponding to biological dosage, demonstrating its capacity to resolve the magnitude of immune modulation rather than just binary states. Data are represented as mean ± SD.

Visualization of the learned latent space separated perturbed from resting states (Figure 4C). Metabolites with strong predicted effects, including indole-3-pyruvic acid, folic acid, 4-methylcatechol, and 2,3-dihydroxy-3-methylbutanoic acid, localized furthest from the DMSO centroid, whereas weak perturbagens, such as homogentisic acid and methionine sulfoxide, remained near the resting-state cluster. We therefore combined Euclidean distance, reflecting perturbation magnitude, and cosine distance, reflecting direction, into a global perturbation score. Parameter sensitivity analysis identified α = 0.25 as the optimal weighting (Figure S5E). Ranking all 364 metabolites using this score identified 148 high-tier metabolites (Figure 4D and Tables S5 and S6). The DPF ranking was broadly concordant with transcriptional perturbation breadth, with perturbation scores strongly correlated with total DEG counts across metabolites (Spearman’s *r* = 0.76, *p* < 2.2 × 10^−16^; Figure 4E). The remaining rank differences may reflect the distinct information captured by the two approaches, as DEG count measures the breadth of significant transcriptional changes, whereas DPF integrates continuous expression patterns associated with T cell state progression. Integrated gradients further identified model-contributing genes related to cytoskeletal remodeling, apoptosis, and mitochondrial respiration, including *COTL1*, *ACTG1*, *ADD3*, *CASP3*, and *NDUFB4* (Figure S6A). Exploratory enrichment of the signed attribution ranking placed Rho GTPase and innate immune programs toward the positive end, and PD-1 and TNF signaling toward the lower end (Figures S6B–S6E). Because these patterns reflect contributions to model output rather than differential expression, they were interpreted as exploratory attribution trends rather than evidence of pathway activation or inhibition.

To test whether this framework generalized beyond the training data, we performed bulk RNA sequencing (RNA-seq) on T cells treated with 12 representative metabolites selected from the active perturbagen group (Table S7). When applied to this independent dataset, DPF consistently assigned higher perturbation scores to metabolite-treated samples than to DMSO controls (Figure 4F). This external validation was supported by concordant upregulation of key activation-associated genes, including *CD3E*, *LCK*, and *JUND*, across these treatment conditions (Figure S7A). From this validated set, we prioritized four candidates, including indole-3-pyruvic acid (IPA), folic acid (FA), hippuric acid (HA), and 4-methylcatechol (4-MC), for in-depth dose-response characterization (1, 10, and 100 μM), selected for their high perturbation scores and low prediction standard deviation. DPF revealed distinct compound-specific concentration-associated trends rather than a uniform response pattern. Hippuric acid and 4-methylcatechol showed increasing mean predicted perturbation scores across the tested concentrations, whereas folic acid and indole-3-pyruvic acid displayed non-monotonic trends, with lower mean scores at 10 μM than at 1 or 100 μM (Figures 4G and S7B and Table S8). Together, these results support the ability of DPF not only to classify perturbation states, but also to rank metabolite activity, generalize across data modalities, and capture quantitative variation across experimental conditions.

### Selected metabolites alleviate DSS-induced acute colitis

Having identified a set of highly active metabolites through perturbation profiling and model-based prioritization, we next asked whether these candidates could also cf. functional benefit *in vivo*. To this end, we selected four representative metabolites, including indole-3-pyruvic acid, folic acid, hippuric acid, and 4-methylcatechol, for evaluation in a dextran sulfate sodium (DSS)-induced acute colitis model (Table S9).

Across all DSS-treated groups, body weight declined over time, with no significant differences among treatment arms (Figure 5A). Despite these similar weight trajectories, clinical disease measures diverged after metabolite administration. Both 4-methylcatechol and folic acid markedly suppressed DSS-induced increases in bloating scores and disease activity index (DAI) compared with the PBS group (Figures 5B and 5C), suggesting an amelioration of intestinal inflammation. Consistent with these clinical improvements, macroscopic examination revealed substantially less colon shortening in mice treated with indole-3-pyruvic acid, folic acid, hippuric acid, and 4-methylcatechol (Figures 5D and 5E).

**Figure 5:**
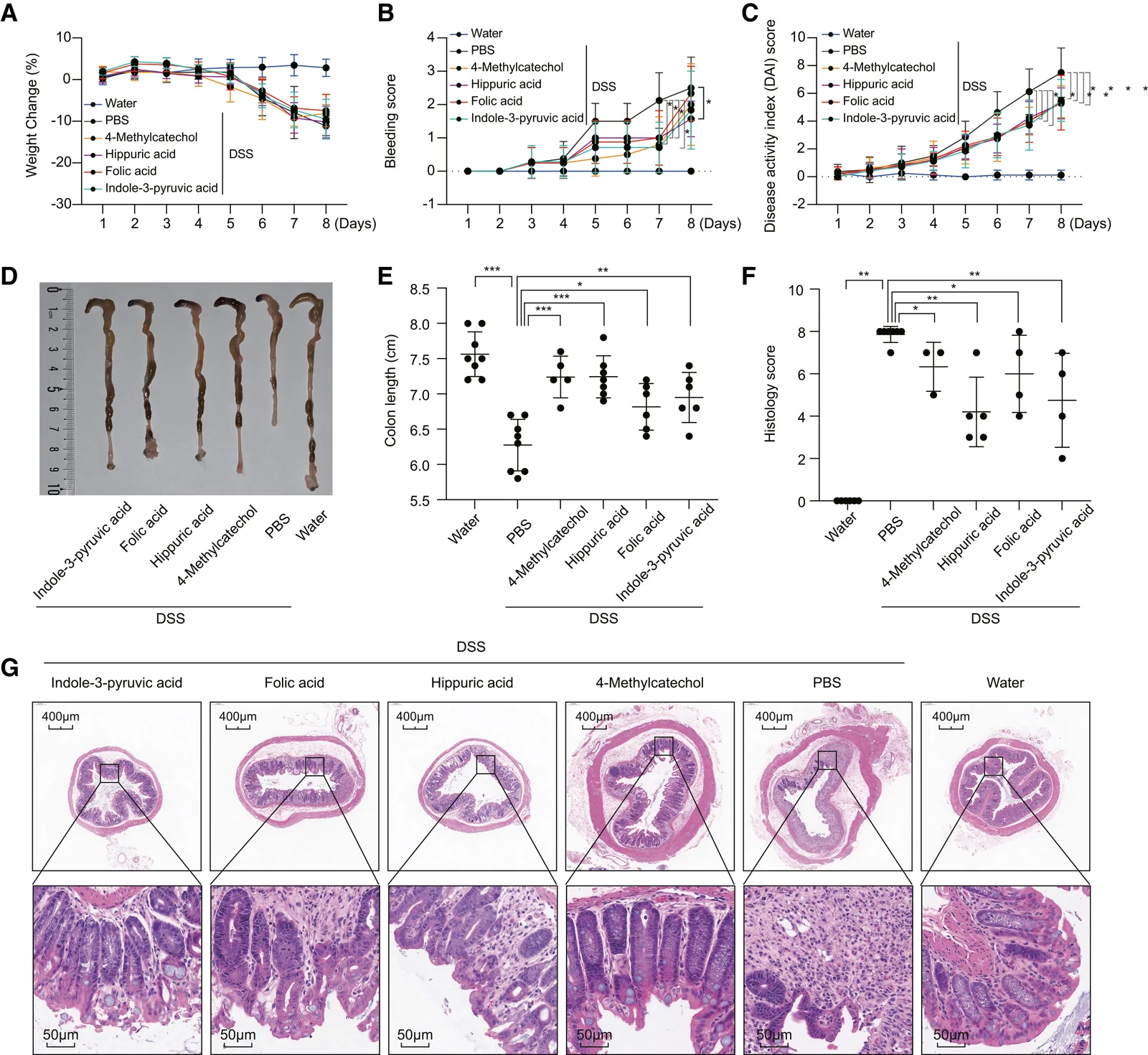
*In vivo* validation of metabolite treatments in DSS-induced colitis (A) Weight change over time in mice treated with DSS, PBS, or various metabolites (4-methylcatechol, hippuric acid, folic acid, and indole-3-pyruvic acid). Data are represented as mean ± standard deviation (SD). (B) Bleeding scores over 8 days in DSS-treated mice receiving metabolite treatments. Data are represented as mean ± SD. Statistical comparisons were performed using Wilcoxon rank-sum tests (∗*p* < 0.05). (C) Disease activity index (DAI) score showing the severity of inflammation in response to different treatments. Data are represented as mean ± SD. Statistical comparisons were performed using Wilcoxon rank-sum tests (∗*p* < 0.05). (D) Representative images of colon length (cm) from each treatment group, showing the effects of metabolite treatments on tissue integrity. (E) Quantification of colon length across treatment groups (represented as mean ± SD), with statistical analysis using two-tailed Student’s *t* tests showing significant differences between the metabolites and control conditions (∗*p* < 0.05, ∗∗*p* < 0.01, and ∗∗∗*p* < 0.001). (F) Histological analysis of colonic tissue sections stained with H&E, demonstrating the effects of metabolites on tissue damage and inflammation in DSS-induced colitis. Data are represented as mean ± SD. Statistical comparisons were performed using Wilcoxon rank-sum tests (∗*p* < 0.05 and ∗∗*p* < 0.01). (G) High-magnification histological images of colonic tissue from different treatment groups, showing the extent of tissue damage and inflammation. Scale bars: 400 μm (top six images) and 50 μm (bottom six images).

Histopathological evaluation further substantiated the protective effects of these metabolites. Compared with PBS controls, colons from mice treated with 4-methylcatechol and folic acid exhibited markedly reduced epithelial damage, crypt destruction, and inflammatory infiltration, resulting in significantly lower histology scores (Figure 5F). H&E staining confirmed preservation of mucosal architecture and reduced inflammatory cell accumulation in these groups (Figure 5G). Indole-3-pyruvic acid and hippuric acid also showed protective trends, with partial improvement in colon length and histopathological features relative to PBS controls. Together, these results indicate that several transcriptionally active metabolites identified *in vitro* can translate into measurable anti-inflammatory effects *in vivo*.

### Metabolite protection *in vivo* is associated with restoration of T cell-related immune programs

To characterize the systemic transcriptional changes accompanying these protective effects, we performed bulk RNA-seq on blood samples from DSS-treated mice receiving metabolite administration (Figure 6A and Table S10). Relative to water controls, DSS+PBS mice showed a pronounced inflammatory shift, characterized by increased expression of *S100a8*, *S100a9*, and *Mmp9* and reduced expression of lymphoid markers such as *Cd79a* (Figure S8A). Consistently, DSS-enriched pathways related to myeloid cell differentiation, leukocyte migration, and cytokine-mediated signaling, while suppressing RNA modification, methylation, and B cell-mediated immunity (Figure S8B).

**Figure 6:**
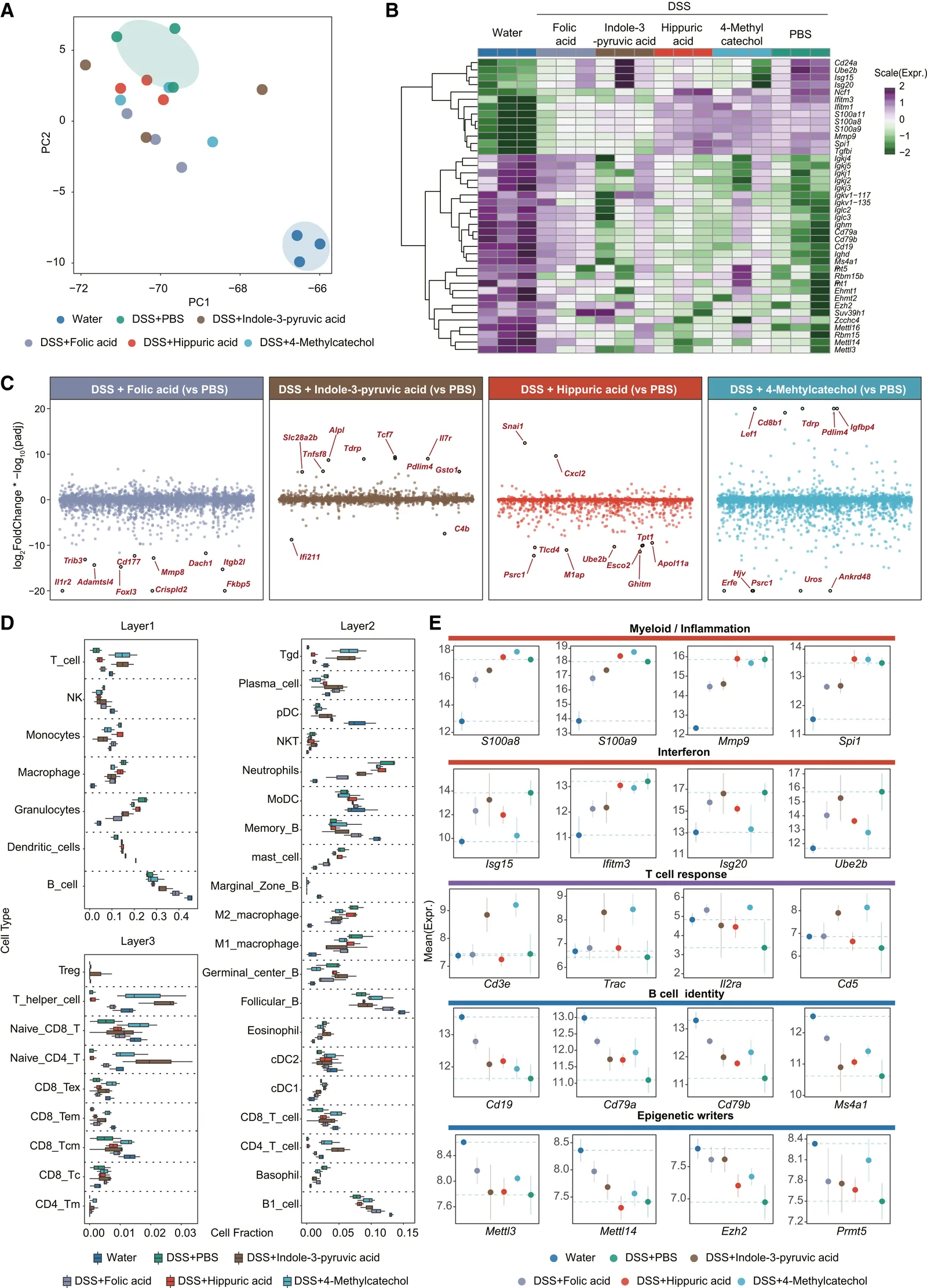
*In vivo* transcriptomic profiling reveals immune cell and inflammatory pathway rewiring following metabolite administration in DSS-induced colitis (A) Principal component analysis (PCA) of colonic RNA-seq profiles from DSS-treated mice administered folic acid, indole-3-pyruvic acid, hippuric acid, or 4-methylcatechol. (B) Heatmap of differentially expressed genes across treatment groups. (C) Scatterplots showing signed differential expression scores for metabolite-treated DSS mice relative to DSS+PBS controls. Scores were calculated as log_2_ fold change × −log_10_(adjusted *p* value). *p* values were calculated using the DESeq2 Wald test and adjusted using the BH method. Representative differentially expressed genes are labeled. (D) Immune deconvolution analysis using the ssGSEA algorithm of ImmuCellAI_mouse, revealing a shift from myeloid-dominant inflammation to a lymphoid-restored landscape. The boxplot displays the distribution of the data. Within each box, the central line represents the median, and the lower and upper hinges of the box denote the 25th (Q1) and 75th (Q3) percentiles. The whiskers extend from the hinges to the largest and smallest values within 1.5 × IQR from the Q1 and Q3, respectively. (E) Expression levels of representative marker genes validating the multi-layered therapeutic mechanism. Boxplots show the downregulation of innate inflammatory drivers (*S100a8*, *S100a9*, *Mmp9*, and *Isg15*) and the restoration of adaptive immunity genes (*Cd3e*, *Trac*, *Il2ra*, and *Cd19*) and critical epigenetic regulators (*Mettl3*, *Mettl14*, and *Ezh2*) following metabolite treatment. Data are represented as mean ± standard deviation (SD).

Metabolite treatment shifted the blood transcriptome away from the DSS-associated inflammatory state. In particular, 4-methylcatechol and folic acid moved closer to water controls in principle component space (Figure 6A). Global and metabolite-specific expression profiles showed reduced interferon-stimulated and innate inflammatory programs, accompanied by partial recovery of adaptive immune and chromatin-regulatory programs (Figures 6B and 6C). Pathway analysis further showed attenuation of inflammatory response and myeloid differentiation programs, together with relative restoration of methylation- and T cell differentiation-related pathways (Figures S8C–S8F). Folic acid showed the broadest reversal pattern, whereas hippuric acid exhibited more partial effects, in line with its intermediate phenotypic protection.

Immune deconvolution supported a coordinated shift from myeloid-dominant inflammation toward lymphoid-associated programs (Figure 6D and Table S11). Treatment with 4-methylcatechol and indole-3-pyruvic acid was associated with higher inferred proportions of Th cells, naive CD4^+^ T cells, and their associated activation score levels, whereas folic acid and hippuric acid were associated with higher inferred proportions of naive and central memory CD8^+^ T cells. In contrast, DSS+PBS animals showed prominent enrichment of neutrophils, inflammatory monocytes, granulocytes, and macrophage-like inflammatory signatures (Figure 6D). These changes were supported at the gene level: myeloid and interferon-response genes, including *S100a8*, *S100a9*, *Mmp9*, *Spi1*, *Isg15*, *Ifitm3*, *Isg20*, and *Ube2b*, were reduced following metabolite treatment, whereas T cell-associated genes (*Cd3e*, *Trac*, and *Il7r*), B cell markers(*Cd19*, *Cd79a*, *Cd79b*, and *Ms4a1*), and epigenetic regulators (*Mettl3*, *Mettl14*, *Ezh2*, and *Prmt5*) showed partial recovery (Figure 6E). Together, these findings indicate that metabolite-mediated protection in DSS colitis is associated with suppression of systemic inflammatory programs and restoration of T cell-related and broader adaptive immune features.

## Discussion

Our results establish a systematic framework for linking metabolite perturbations to T cell state transitions, predictive modeling, and *in vivo* functional validation. The gut microbiome and host metabolome together generate a large and chemically diverse pool of small molecules that can shape immune behavior, yet metabolite-immune relationships have been difficult to define systematically with conventional low-throughput approaches. Here we address this gap by combining high-throughput perturbation profiling with predictive modeling to map how metabolites reshape transcriptional programs in human T cells. Using DRUG-seq, we profiled 364 endogenous and microbiota-associated metabolites and organized their effects into a structured transcriptional state space. We then used a multi-task transformer model, DPF, to quantify perturbation strength, prioritize candidate immunomodulators, and guide *in vivo* validation, which we demonstrated in a DSS-induced acute colitis model. This integrated framework provides both an interpretable atlas of metabolite-induced programs and a scalable route to nominate candidates for downstream functional testing.

A central contribution of the atlas is that it establishes a shared transcriptional reference enabling direct comparison across chemically diverse metabolites. Within this reference, well-studied metabolite classes such as SCFAs, indole derivatives, and bile-acid-related metabolites aligned with activation-associated or cytokine-responsive transcriptional programs, supporting the biological fidelity of the platform. Equally informative, we identified high-impact perturbagens outside these canonical families, including 2-methylbutanoic acid, tocotrienol, and resveratrol, together with indole-3-pyruvic acid, indicating that immunometabolic regulation is not restricted to the metabolite categories most frequently studied. Notably, these metabolites converged on a coherent activation-like output, exemplified by induction of *IL2RA* and proximal signaling components such as *LCK*, suggesting that diverse metabolites can funnel into shared T cell activation circuitry rather than generating unrelated signatures. In this way, our dataset complements earlier high-throughput perturbation efforts such as DRUG-seq,^19^^,^^20^ while extending deep perturbation learning concepts into the metabolite domain.^23^^,^^24^

Beyond cataloging responses, the screen revealed that metabolite effects are recurrent and modular. Rather than producing diffuse and idiosyncratic signatures, metabolites concentrated into discrete transcriptional programs that could be interpreted as coordinated activation of metabolic pathways and TCR-associated signaling modules.^25^ These programs were anchored by interpretable molecular features, with the TCR-high activation state tracking upregulation of core T cell signaling and activation genes such as *CD3E*, *CD247*, *LCK*, and *IL2RA*, while the metabolic active state preferentially engaged mitochondrial and bioenergetic genes including *NDUFA11* and *NDUFB8*. This anchoring makes the state space actionable by directly linking metabolite ranking to testable pathway hypotheses, rather than to abstract clustering labels. These programs define a continuum of states spanning baseline-like profiles through metabolically activated intermediates to highly responsive endpoints, suggesting that metabolites can shift T cells along an activation trajectory in a graded manner. Along this continuum, *STAT3* and *KLF2* mark baseline-like programs, whereas *ATF4* and *JUND* track progression toward metabolically activated and highly responsive states, providing practical molecular landmarks for mechanistic follow-up. This framing provides a practical way to compare metabolites not only by the number of responsive genes, but by the directionality and state identity of the programs they induce.

Trajectory inference and regulon analysis provided additional context for these state transitions and highlighted regulatory nodes associated with different portions of the continuum. The inferred hierarchy highlights concrete candidate control points for mechanistic testing, with baseline-associated regulators such as *STAT3* and *KLF2* and terminal modules involving *ATF4* and *JUND*, alongside *RELA* within the high-response program. This pattern is consistent with known requirements for T cells to acquire metabolic fitness to function in nutrient-limited inflammatory settings.^26^ Along the inferred trajectory, *STAT3* activity decreased in parallel with induction of metabolic programs, including upregulation of mitochondrial genes such as *NDUFB8*.^27^ In contrast, *IRF1* remained relatively low during early MA, a pattern that may constrain interferon-associated transcriptional programs while metabolic capacity is being established.^28^ These observations support a staged model in which metabolic licensing and inflammatory transcription can be partially uncoupled, although direct causality will require targeted perturbation of these regulators and upstream sensors.

Changes in subtype-associated transcriptional signatures further suggest that metabolite-driven programs are coupled to functional remodeling. Central memory signatures increased across states, consistent with the possibility that some metabolites promote metabolic competence while preserving features linked to long-term persistence.^29^ Conversely, CD4-associated effector-memory signatures declined while CD8-associated effector-memory signatures increased, indicating non-uniform remodeling across CD4 and CD8 compartments. Because these are signature-based inferences rather than lineage-tracing measurements, they are best interpreted as shifts in program utilization rather than definitive differentiation pathways. Nonetheless, they reinforce that metabolite perturbations do not simply amplify generic activation, but reshape the balance of functional programs across T cell states. The gradual increase in Th17 signatures with relatively stable Th1 signatures similarly points to selective remodeling. This trend is compatible with established metabolic dependencies of Th17 differentiation and RORγt-associated programs,^30^ and it motivates focused testing of whether top perturbagens (e.g., folic acid) preferentially stabilize Th17-linked transcriptional modules under defined conditions.

A second major contribution of this study is the DPF model, which achieved strong performance in predicting metabolite-induced transcriptional perturbations (AUC = 0.987). Recent transcriptome-scale modeling studies have shown that self-attention-based architectures can learn high-order gene-gene relationships and shared regulatory patterns from high-dimensional expression data, providing a general framework for inference across perturbations and cellular contexts.^31^^,^^32^^,^^33^ Within this landscape, DPF functions as a practical prioritization layer for metabolite perturbation data: it integrates multi-task outputs that reflect activation depth and state progression, enabling systematic ranking of candidates from a large chemical space and supporting inference beyond experimentally assayed conditions. The biological relevance of this ranking was supported by the presence of well-characterized immunomodulatory metabolites within the high tier. These included histamine, which shapes T cell polarization^34^; ATP and succinate, which connect extracellular and metabolic cues to Th17 differentiation and inflammatory signaling^35^^,^^36^; and indole-3-carboxaldehyde and 3-hydroxyanthranilic acid, which regulate mucosal AhR-IL-22 signaling and activated T cell responses,^37^^,^^38^ respectively. These established signals anchor the DPF ranking in known immunometabolic biology, while the broader high-tier set extends prioritization to less-characterized candidates.

Importantly, the model’s utility is not limited to assigning a “perturbed” or “resting” label; it also captures compound-specific dynamics that can inform experimental design. In our dataset, DPF-ranked candidates such as indole-3-pyruvic acid and folic acid clustered at the extreme end of the perturbed manifold, consistent with large Euclidean distances from DMSO controls and concordant induction of activation modules. In follow-up concentration profiling of prioritized candidates, we observed distinct concentration-associated trends across metabolites, including apparent non-monotonic trends for folic acid and indole-3-pyruvic acid. These divergent patterns may reflect compound-specific concentration-dependent behavior potentially related to receptor-mediated sensing pathways, such as AhR-dependent signaling, or from concentration-dependent engagement of core metabolic processes, including one-carbon metabolism.^39^^,^^40^^,^^41^ Further profiling across a broader and more densely sampled concentration range will be important for defining the reproducibility and biological basis of these patterns.

The value of this prediction-to-validation loop was supported by *in vivo* testing. Representative DPF-prioritized metabolites were evaluated in a DSS-induced acute colitis model^42^; all four metabolites, 4-methylcatechol, folic acid, hippuric acid, and indole-3-pyruvic acid, showed measurable protection, reflected by lower disease activity, reduced colon shortening, and improved histopathology compared with DSS+PBS controls, supporting their potential as modulators of intestinal inflammation. These phenotypic outcomes firmly establish their *in vivo* efficacy but remain mechanistically agnostic. Several plausible upstream sensors have been proposed in previous studies, such as Nrf2-Keap1/NF-κB axes for 4-methylcatechol,^43^^,^^44^^,^^45^ AhR signaling for indole-3-pyruvic acid,^46^^,^^47^^,^^48^ one-carbon metabolism/MTHFD2 for folic acid,^49^^,^^50^^,^^51^^,^^52^ and context-dependent immunoregulation for hippuric acid.^53^ However, because none of these specific pathways were directly perturbed or pharmacologically validated in our current experiments, we regard these connections as hypothetical frameworks to guide future mechanistic dissection, rather than as definitive conclusions drawn from our study.

More importantly, our transcriptomic data revealed a convergent downstream output across the effective metabolites, namely a global transcriptional shift consistent with suppression of myeloid-driven inflammatory programs.^54^ We also observed restoration of expression patterns involving epigenetic regulators such as *Mettl3*,^55^ and across four validated anti-inflammatory metabolites, a shared signature consistent with re-engagement of the *METTL3/14* and *EZH2* regulatory axis emerged. While these findings nominate epigenetic regulation as a plausible convergence point, establishing whether these changes reflect direct, causal remodeling of chromatin or RNA modification will require orthogonal measurements at the protein and chromatin levels. Nonetheless, this shared transcriptional rebalancing suggests that these chemically diverse metabolites may share common state-regulatory programs, rather than operating through entirely distinct linear pathways implicated by previous researches. Defining whether the proposed upstream sensors that actually mediate these effects, and how they couple to the epigenetic and transcriptional shifts represents a prioritized avenue for subsequent mechanistic studies.

In summary, by integrating systematic perturbation profiling, deep-learning-based prioritization, and *in vivo* validation, this study delivers a scalable framework for mapping immunometabolic regulation. The atlas and DPF model together provide an experimentally grounded and computationally actionable foundation to identify metabolites that reshape T cell states, clarify regulatory programs underlying these transitions, and nominate candidates with translational potential.

### Limitations of the study

Our study provides an experimentally grounded atlas and a practical prediction-to-validation framework for metabolite-driven T cell programs, and it also highlights several areas where additional work will further strengthen interpretation and broaden applicability. Because the atlas was generated in a standardized *in vitro* system using activated human T cells, it necessarily abstracts away some elements of the physiological environments in which metabolites act, including dynamic nutrient availability, cytokine gradients, and multicellular crosstalk with epithelial, stromal, and myeloid compartments. To enable systematic comparison across the metabolite library, all 364 metabolites were screened at a uniform concentration of 4 μM, providing a common reference for identifying and ranking metabolite-induced transcriptional perturbations. Because physiological abundance, tissue exposure, bioavailability, and dose-response relationships differ among metabolites, these rankings are best interpreted as relative perturbation strengths under the defined screening conditions. Extension to larger and more diverse donor cohorts, together with concentration-response profiling across physiologically relevant ranges, will further establish the generalizability of the identified metabolite-responsive programs and refine the biological interpretation of individual metabolites.

The transcriptome offers a sensitive and scalable readout of state remodeling, but translating transcriptional programs into definitive signaling, protein-level regulation, and functional phenotypes remains a recognized challenge in immunometabolic studies, making state-resolved functional characterization an important direction for our future work. Building on the transcriptional framework established here, our future work will therefore focus on systematic state-resolved functional characterization to determine how these molecular programs shape distinct T cell phenotypes and immune functions. Along similar lines, regulatory features suggested by trajectory and regulon analyses, as well as the apparent convergence on epigenetic regulators such as *METTL3/14* and *EZH2*, are supported by consistent expression patterns in our data and represent strong mechanistic hypotheses. Establishing direct causality requires targeted perturbation experiments and orthogonal measurements at the chromatin and protein levels, which can be technically demanding in primary T cells. The DPF model achieves high performance within the chemical and biological space represented in training data, yet continued testing in additional activation contexts and across immune compartments will be important to define the scope of generalization for untested metabolites. Moreover, our *in vivo* validation was limited to a small set of prioritized metabolites in a DSS-induced colitis model and was intended as a proof of concept. Because the discovery screen was performed in activated human T cells whereas validation was conducted in mice, the two components provide complementary rather than directly equivalent evidence. Species-specific differences in immune organization, metabolite disposition, tissue exposure, microbiota, and inflammatory context may influence the observed responses. The exclusive use of male mice may further limit generalizability, given the established influence of sex in IBD (Inflammatory Bowel Disease) models. Future studies in human-relevant systems, both sexes, and additional inflammatory or malignant settings, together with characterization of proximal sensors and pharmacological constraints, will be needed to assess the translational potential of these findings.

## Resource availability

### Lead contact

Requests for further information and resources should be directed to and will be fulfilled by the lead contact,Yun Tan (ty12260@rjh.com.cn).

### Materials availability

This study did not generate new unique reagents.

### Data and code availability

• All raw sequencing data are accessible in the National Omics Data Encyclopedia (NODE: OPE000006828)https://www.biosino.org/node: https://www.biosino.org/node/project/detail/OEP00006828. Processed bulk RNA-seq gene-level count matrices have been deposited at Zenodo:https://doi.org/10.5281/zenodo.18266499). An interactive web portal for DRUG-seq data exploration is available at https://www.transmed.sh.cn.
• The source code used in this study has been archived at Zenodo: https://doi.org/10.5281/zenodo.18308150). The actively maintained development version is available at https://github.com/Bingobin/DRUGseq-PerturbFormer.
• Any additional information required to reanalyze the data reported in this paper is available from the lead contact upon request.

## Acknowledgments

This study is funded by the 10.13039/501100001809National Natural Science Foundation of China (grant nos. 82370154, 82230006, 82100158, 82300154, and 82200155), the Noncommunicable Chronic Diseases-10.13039/501100018537National Science and Technology Major Project (grant nos. 2026ZD0553900, 2023ZD0500700), and the Natural Science Foundation of Shanghai (grant nos. 24QA2705100, 21ZR1480900, and 21YF1427900). We thank the support from the High-throughput Drug Screening Platform, Sequencing Platform, and Bioinformatic Platform of the National Facility for Translational Medicine (Shanghai). The computations in this paper were run on the Siyuan-1 cluster supported by the Center for High Performance Computing at Shanghai Jiao Tong University.

## Author contributions

Y.X., Y.Z., and S. Yang contributed equally to the study. Y.T. designed this study. Y.L. performed the bioinformatic analysis. Y.X., Y.Z., S. Yang, H.L., and X.D. performed most experiments. Y.X., Y.T., and Y.L. drafted the manuscript. Y.T. and Y.L. revised the manuscript. Y.Z., S. Yang, H.L., X.D., Q.Z., S. Yu, S.W., F.J., Q.W., Y.M., and B.J. interpreted the data, made critical revision of the manuscript for important intellectual content, and approved the final submitted version of the manuscript.

## Declaration of interests

The authors declare no competing interests.

## STAR★Methods

### Key resources table

**Table:** 

REAGENT or RESOURCE	SOURCE	IDENTIFIER
**Chemicals, peptides, and recombinant proteins**		

X-VIVO® 15	Lonza	Cat# 04-418Q
FBS	Vazyme	Cat# F102-01
RNA isolater Total RNA Extraction Reagent	Vazyme	Cat# R401-01
Human Recombinant Interleukin 2	STEMCELL Technologies	Cat# 78036
T cell TransAct(TM), human	Miltenyi Biotec	Cat# 130-128-758
Dextran sulfate sodium salt (DSS, MW 4500-5500)	TargetMol	Cat# T13647
4-Methylcatechol	TargetMol	Cat# T4779
Hippuric acid	TargetMol	Cat# T4815
Folic acid	TargetMol	Cat# T0062
Indole-3-pyruvic acid	TargetMol	Cat# T4939

**Critical commercial assays**		

EasySep™ Human CD3 Positive Selection Kit II	STEMCELL Technologies	Cat# 17851
Mini Total RNA Extraction Kit	Jianshi Biotechnology	Cat# TR205
Fast CE RNA-seq Lib Prep Kit	ABclonal	Cat# RK20311

**Deposited data**		

Human reference genome, GRCh38	Genome Reference Consortium	https://www.ncbi.nlm.nih.gov/grc/human
Mouse reference genome, GRCm39	Genome Reference Consortium	https://www.ncbi.nlm.nih.gov/grc/mouse
Raw data	This paper	https://www.biosino.org/node/project/detail/OEP00006828
Processed bulk RNA-seq gene-level count matrices	This paper	https://doi.org/10.5281/zenodo.18266499
An interactive web portal for DRUG-seq data exploration	This paper	https://www.transmed.sh.cn

**Experimental models: Organisms/strains**		

Male C57BL/6J mice(7-9 weeks, ♂)	Shanghai Model Organisms	RRID:IMSR_JAX:000664

**Software and algorithms**		

FastQC v0.12.1	Babraham Bioinformatics	https://www.bioinformatics.babraham.ac.uk/projects/fastqc/
UMI-tools v1.1.2	Smith et al.^56^	https://github.com/CGATOxford/UMI-tools
Fastp v0.23.2	Chen et al.^57^	https://github.com/OpenGene/fastp
STAR v2.7.11b	Dobin et al.^58^	https://github.com/alexdobin/STAR
featureCounts v2.0.1	Liao et al.^59^	https://subread.sourceforge.net/
SAMtools v1.21	Li et al.^60^	https://www.htslib.org/
Seurat v5.0	Hao et al.^61^	https://satijalab.org/seurat/
DESeq2 v1.46.0	Love et al.^62^	https://bioconductor.org/packages/DESeq2/
clusterProfiler v4.14.6	Xu et al.^63^	https://bioconductor.org/packages/clusterProfiler/
pySCENIC v0.12.1	Aibar et al.^64^	https://github.com/aertslab/pySCENIC
GSVA v2.4.2	Hänzelmann et al.^65^	https://bioconductor.org/packages/GSVA/
Monocle3 v1.4.26	Qiu et al.^66^	https://cole-trapnell-lab.github.io/monocle3/
CytoTRACE2 v1.1.0	Kang et al.^67^	https://github.com/digitalcytometry/cytotrace2
ImmuCellAI-mouse	Miao et al.^68^	https://guolab.wchscu.cn/ImmuCellAI-mouse/#!/
DRUGseq-PerturbFormer	This paper	https://doi.org/10.5281/zenodo.18308150; https://github.com/Bingobin/DRUGseq-PerturbFormer

### Experimental model and study participant details

#### Animals

Healthy, 7-9-week-old male C57BL/6J mice were purchased from Shanghai Model Organisms (Shanghai, China). All mice were housed under specific-pathogen-free conditions with 50±5% humidity at 22±2°C with a 12/12 h light/dark cycle.

#### Ethics statement

This study was approved by the Ethics Committee of Ruijin Hospital, Shanghai Jiao Tong University School of Medicine. Animal experiments were performed in accordance with guidelines approved by the Institutional Animal Care and Use Committee (IACUC) of Ruijin Hospital, Shanghai Jiao Tong University School of Medicine (approval number: RJ2026011). Informed consents had been obtained from all the enrolled donors. All experiments were conducted in accordance with relevant guidelines and regulations, ensuring compliance with ethical standards for the use of human subjects, with the ethical approval number 2025-717.

### Method details

#### T cells extraction and drug treatment

To mitigate the impact of individual variability on the analytical results, peripheral blood mononuclear cells (PBMCs) were isolated from peripheral blood obtained from two healthy volunteers (male, aged from 21 to 29) using density gradient centrifugation. T cells were purified from PBMCs using the EasySep™ Human CD3 Positive Selection Kit II (STEMCELL Technologies, 17851) and cultured in X-VIVO® 15 medium (Lonza, 04-418Q) supplemented with 5% FBS (Vazyme, F102-01) and 400 U/mL Human Recombinant Interleukin 2 (STEMCELL Technologies, 78036). To induce activation, T cells were stimulated with T Cell TransAct™ (Miltenyi Biotec, 130-128-758) for 48 hours.

Following activation, the cell suspension was adjusted to 1 × 10^6^ cells/mL, and 50 μL aliquots were dispensed into 384-well cell culture plates using a MultiDrop Combi dispenser (ThermoFisher, 5840300). A pre-configured compound library containing 364 metabolites and 20 DMSO controls was utilized for the screen. Metabolites (200 nL/well) were transferred using an Explorer G3 workstation (PerkinElmer) to achieve a final concentration of 4 μM. This uniform concentration was selected to enable standardized high-throughput screening and direct comparison across the metabolite library, while balancing the detection of biological effects with minimal cytotoxicity. Although it may exceed physiological circulating levels for certain metabolites, it was intended as a common screening condition rather than to reproduce the physiological exposure of each individual metabolite.

#### Cell lysis, library construction and sequencing for DRUG-seq

Fourty-eight hours after drug treatment, cells in each well were centrifuged at 2000 rpm for 10 minutes, and the supernatant was removed. Then, cell lysis and mRNA library construction for the T cells in each well were performed using a modified DRUG-seq library preparation method, starting with reverse transcription of cell lysates using barcoded primers, followed by pooled purification and exonuclease treatment to remove unwanted nucleic acids. cDNA is then amplified by PCR and purified. An aliquot of the preamplified material is tagmented, purified, and subjected to a second PCR with indexing primers. The final libraries are purified, size-selected for 200-600 bp fragments, and assessed for concentration and average size (∼300 bp) before sequencing.^19^^,^^69^ To uniquely identify transcripts from all 384 wells, a combinatorial indexing strategy was employed, utilizing 96 well-specific barcodes introduced during reverse transcription and four plate-specific indices added during the final PCR amplification.

Library size distribution and concentration were assessed using the 4200 TapeStation (Agilent Technologies) and Qubit 3 Fluorometer (Thermo Fisher Scientific), respectively. After confirming the fragmentation profile and obtaining quantitative data for each library, libraries were pooled in proportion to their concentrations and subjected to sequencing on the MGISEQ-2000 platform (MGI).

#### RNA extraction and library construction for bulk RNA-seq

Mouse peripheral blood and T cells were lysed in RNA isolator Total RNA Extraction Reagent (Vazyme, R401-01). We used Mini Total RNA Extraction Kit (Jianshi Biotechnology, TR205) for total RNA extraction and Fast CE RNA-seq Lib Prep Kit (ABclonal, RK20311) for cDNA library synthesis according to the manufacturer’s instructions. The following steps are the same as above.

#### Animals and treatment

The mice were randomly divided into six groups on average: blank control group, model control group, and four treatment groups. Blank control mice received normal drinking water. For the DSS-induced colitis model, mice were administered with 2.5% Dextran sulfate sodium salt (MW 4500-5500, TargetMol, T13647) in drinking water for 6 days, followed by 2 days of regular drinking water. We obtained four types of drug powders: 4-Methylcatechol (TargetMol, T4779), Hippuric acid (TargetMol, T4815), Folic acid (TargetMol, T0062), and Indole-3-pyruvic acid (TargetMol, T4939). Four treatment groups were intraperitoneally injected with a certain dose (4-Methylcatechol: 20mg/kg mouse weight; Hippuric acid: 3mg/kg mouse weight; Folic acid: 20mg/kg mouse weight; Indole-3-pyruvic acid: 20mg/kg mouse weight) of drug solution dissolved in PBS every day, while the blank control group and the model control group were injected with an equal volume of PBS every day for solvent control. The dose of hippuric acid was limited by its poor solubility, while the other three metabolites could be readily formulated in PBS at higher concentrations and were therefore administered at a common exploratory dose of 20 mg/kg/day, chosen to provide a consistent proof-of-concept condition across these compounds informed by prior *in vivo* use^70^^,^^71^^,^^72^ where available.

The mice were sacrificed on day 8. Body weight loss, rectal bleeding, and diarrhea were monitored and recorded daily from Day 0 to Day 8. The scoring criteria were as follows: Weight loss: 0, none; 1, 1-5%; 2, 5-10%; 3, 10-20%; 4, >20%. Stool consistency: 0, well-formed pellets; 1, semisolid and not adherent to the anus; 2, semiformed and adherent to the anus; 4, liquid stools adherent to the anus with diarrhea. Bleeding score: 0, no blood in stool; 1, occult or faint blood; 2, clearly visible blood; 4, gross rectal bleeding. The Disease Activity Index (DAI) score for each mouse was calculated by summing the scores for weight loss, stool consistency, and bleeding to assess overall disease severity.^73^

#### Histological analysis

Colon tissues were fixed by immersion in a solution containing 4% paraformaldehyde and subsequently paraffin-embedded. Sections of 5 μm thickness were prepared, deparaffinized, rehydrated, and analyzed using hematoxylin and eosin (H&E) staining to evaluate the extent of colon damage and inflammation. Histological alterations were assessed using a cumulative scoring system. Four scores were assigned based on the degree of epithelial damage: 0, normal morphology; 1, loss of goblet cells; 2, loss of goblet cells in large regions; 3, loss of crypts; 4, loss of crypts in large areas. Additionally, the degree of inflammatory cell infiltration was graded as follows: 0, no infiltration; 1, infiltration around the crypt base; 2, infiltration extending to the muscularis mucosa; 3, extensive infiltration into the muscularis mucosa accompanied by significant edema; 4, infiltration spreading to the submucosa.^73^

#### Bioinformatics workflow

##### Processing of raw reads for DRUG-seq

The raw paired-end sequencing data obtained from the DRUG-seq experiments were processed using a custom bioinformatics pipeline. Initially, data quality was assessed using FastQC (v0.12.1). To ensure accurate well-based demultiplexing, a comprehensive barcode whitelist was generated using a custom script, allowing for a one-base pair mismatch tolerance. Well barcodes and Unique Molecular Identifiers (UMIs) were extracted from the first read using UMI-tools (v1.1.2) based on this whitelist, with cell barcode error correction enabled. The reads were subsequently trimmed and filtered using Fastp (v0.23.2)^57^ to remove adapter sequences and low-quality bases. The resulting clean reads were aligned to the human reference genome (GRCh38/hg38) using the STAR aligner (v2.7.11b). To quantify gene expression, featureCounts (v2.0.1)^59^ was employed to assign mapped reads to genomic features using the -R BAM parameter, which tags aligned reads with gene assignment information. Finally, the gene-tagged BAM files were sorted using SAMtools (v1.21),^74^ and UMI-tools count was utilized to deduplicate PCR artifacts and generate a digital gene expression matrix by counting unique molecules per gene for each well.

##### DRUG-seq transcriptomic analysis

The digital gene expression matrices generated by UMI-tools were processed and analyzed using the Seurat R package (v5.0).^61^ The two donor-derived screens comprised eight 96-well plates in total, with each plate containing 91 metabolite-treated wells and five DMSO-control wells (Table S1). To ensure data quality, wells with fewer than 150 detected Unique Molecular Identifiers (UMIs) were excluded from downstream analysis. To reduce plate-associated technical variation during dimensionality reduction and clustering, the RNA assay was split by physical plate, and each of the eight plate-level datasets was independently normalized and variance-stabilized using SCTransform with the glmGamPoi implementation and 3,000 variable features. Principal component analysis was performed on the SCT-normalized data, followed by integration of the plate-level datasets using reciprocal PCA. The resulting integrated representation was used for UMAP visualization and unsupervised clustering based on the shared nearest-neighbor graph. Cluster-specific marker genes were identified using the FindAllMarkers function on the SCT-normalized assay, with significance criteria set at an adjusted *p*-value < 0.05, an average log_2_ fold change > 1, and a minimum expression in 25% of wells within the cluster. Functional annotation of these markers was conducted using the clusterProfiler R package (v4.14.6)^63^ to identify enriched KEGG pathways (Benjamini-Hochberg adjusted *p* < 0.05). To define the transcriptomic baseline representing the homeostatic state, we evaluated the distribution of control samples (e.g., DMSO-treated wells) across the identified clusters; a Fisher’s exact test was applied to each cluster, and clusters showing a statistically significant enrichment of control wells (*p* < 0.001) were designated as the “Baseline” state, serving as the reference for characterizing drug-induced perturbations.

##### Identification of metabolite-induced transcriptomic perturbations

Iterative pairwise differential expression analyses were performed using the DESeq2 R package (v1.46.0)^62^ to characterize metabolite-induced transcriptional changes. Each metabolite-treated group was compared specifically against its corresponding intra-plate DMSO controls using raw UMI count matrices. This comparison-specific design was adopted to minimize plate-associated technical variation while preserving metabolite-specific transcriptional perturbation patterns. Genes with no expression were pre-filtered. Differentially expressed genes (DEGs) were identified based on a threshold of adjusted *p*-value < 0.05 and |log_2_ fold change| > log_2_(1.5). The lists of significantly up- or downregulated genes from all metabolite-specific comparisons were combined by union to construct the “Perturbed Gene Universe” (*N* = 2,541). This gene set represents an inclusive collection of genes responsive to at least one metabolite perturbation and was used as the feature space for downstream DPF model training. Because multiple-testing correction was performed separately within each metabolite-versus-DMSO comparison, the Perturbed Gene Universe should not be interpreted as a globally FDR-controlled catalogue of DEGs across all gene-metabolite tests. Functional states were quantified using Seurat's AddModuleScore: a T cell activation Score (Tscore) and Energy-metabolic activation score (MAscore) were calculated based on relevant GO gene sets, while a TCR Score (TCRscore) was derived from the expression of *TRBC1*, *TRBC2*, and *TRAC*.

##### Functional dynamics and regulatory network inference

To unravel regulatory mechanisms, transcription factor regulon activity was inferred using pySCENIC (v0.12.1),^64^ with Regulon Activity Scores (RAS) calculated based on co-expression modules. The relative abundance of 13 distinct T cell subtypes was estimated via ssGSEA using GSVA (v2.4.2)^65^ on VST-normalized data (DESeq2). To model differentiation trajectories, Monocle3 (v1.4.26)^66^ was utilized. UMAP coordinates from Seurat were imported directly to construct the trajectory graph. Cells from the Resting Cluster (Cluster 6) were designated as the root to calculate pseudotime. Additionally, cellular differentiation potential was independently assessed using CytoTRACE2 (v1.1.0)^67^ applied to the raw count matrix.

##### Deep learning modeling of transcriptomic perturbations

To predict phenotypic states and quantify the magnitude of metabolite-induced perturbations, we developed a multi-task deep learning framework named DRUG-seq-PerturbFormer (DPF). The model utilizes the VST-normalized expression profiles of the 2,541 perturbed genes (identified in the differential analysis step) as input features. We employed a gene-wise tokenization strategy where each gene is projected into a learnable embedding space and processed by a Transformer Encoder (hidden dimension = 128, attention heads = 4) to capture complex gene-gene interactions, resulting in a compact latent embedding (dimension = 16) for each well. The model was trained using a multi-task learning objective to simultaneously optimize the prediction of diverse phenotypic attributes: (1) regression of the three continuous functional scores (Tscore, CytoTRACE2, and Pseudotime) using Mean Squared Error (MSE) loss; and (2) binary classification of cell states (Resting vs. Perturbed) using Binary Cross-Entropy (BCE) loss. The total loss function was defined as the sum of these components, and the model was optimized using the AdamW optimizer (learning rate = 1 × 10^-3^, batch size = 32) for 50 epochs with an 80/20 train-validation split.

To construct a robust metric for ranking metabolite potency, we integrated two distance metrics derived from the latent space: Euclidean distance (representing perturbation magnitude) and Cosine distance (representing directional shift) relative to the DMSO centroid. A unified PerturbScore was defined as a weighted sum: *PerturbScore*= α ×*d*^(*Euc*)^+ (1 - α) ×*d*^(*Cos*)^.The optimal weight *α*=0.25 was determined via a grid search strategy (step size 0.01) by maximizing the separation efficiency between Perturbed and Resting states, evaluated using the Area Under the Curve (AUC), Kolmogorov-Smirnov (KS) statistic, and Cohen’s D effect size. Metabolites were stratified into three tiers based on the empirical distribution of DMSO control scores (*S*_*DMSO*_). Let *Q*_*p*_ denote the *p*-th percentile of *S*_*DMSO*_. The tiers were defined as: High-perturbation (*S*>*Q*_0.75_), Mid- perturbation (*Q*_0.5_<*S*<*Q*_0.75_), and Low-perturbation (*S*<*Q*_0.75_). The source code is available at https://github.com/Bingobin/DRUGseq-PerturbFormer.

Gene-level contributions to the DPF-derived perturbation score were estimated using Integrated Gradients. For each sample, attributions were calculated relative to four plate-level DMSO reference profiles covering all 16 C6 DMSO samples. The attribution values obtained from the four baselines were averaged for each gene and then normalized within each sample by the sum of the absolute attribution values across all 2,541 input genes. The mean signed Integrated Gradient value for each gene was calculated by averaging its normalized signed attribution across all analyzed samples. Positive and negative values therefore indicate contributions toward higher and lower model-derived perturbation scores, respectively. Genes were ranked by the mean signed attribution, and the complete ranked list was used for exploratory Reactome gene-set enrichment analysis.

##### Bulk RNA-seq data processing and analysis

Raw sequencing reads were quality-controlled and filtered using FastQC (v0.12.1) and Fastp (v0.23.2),^57^ then aligned to the *Mus musculus* genome (GRCm39) using STAR (v2.7.11b). Gene expression was quantified by featureCounts (v2.0.1).^59^ Differential expression analysis was performed using DESeq2 (v1.46.0)^62^ to compare DSS vs. BLANK and drug-treated groups vs. DSS. Significant DEGs were identified (adj. *p* < 0.05, |log_2_FC| > log_2_(1.5)). Gene Set Enrichment Analysis (GSEA) was conducted on the ranked gene lists, while Gene Ontology (GO) over-representation analysis was performed separately for significantly upregulated and downregulated genes using clusterProfiler (v4.14.6),^63^ with terms considered significant at an adjusted *p* value < 0.05. Finally, the relative abundance of infiltrating immune cells was estimated from TPM profiles using ImmuCellAI-mouse.^68^

### Quantification and statistical analysis

All statistical analyses were performed using R (v4.2.1). Detailed descriptions of the statistical tests used are provided in the corresponding sections, figure legends, and supplementary tables. Technical reproducibility of the DRUG-seq experiments was assessed using two independent experimental batches. Bulk RNA-seq analyses and mouse validation experiments were conducted with at least three independent biological replicates, as specified in Tables S7, S8, S9, and S10.

## Supplemental information

Document S1. Figures S1–S8

Table S1. Quality control metrics and metadata for DRUG-seq profiling of 364 metabolites

Table S2. Cluster-specific marker genes for the eight identified T cell transcriptional states

Table S3. KEGG pathway enrichment analysis of genes defining T cell transcriptional clusters

Table S4. Differential gene expression summary and state classification for each metabolite treatment

Table S5. Summary of DPF-derived perturbation metrics for all metabolite treatments

Table S6. DPF perturbation scores and global rankings for the 148 high-tier metabolites

Table S7. Metadata for *in vitro* bulk RNA-seq of CD3^+^ T cells derived from human PBMCs

Table S8. Summary of the dose-response bulk RNA-seq validation experiment

Table S9. Clinical and histopathological outcomes in the DSS-induced acute colitis model

Table S10. Metadata for *in vivo* bulk RNA-seq in the DSS-induced acute colitis model

Table S11. Immune cell deconvolution analysis of blood samples from DSS-treated mice

## References

[1] Rooks M.G., Garrett W.S. (2016). Gut microbiota, metabolites and host immunity. Nat. Rev. Immunol..

[2] Takeuchi T., Nakanishi Y., Ohno H. (2024). Microbial Metabolites and Gut Immunology. Annu. Rev. Immunol..

[3] Xie J., Liu M., Deng X., Tang Y., Zheng S., Ou X., Tang H., Xie X., Wu M., Zou Y. (2024). Gut microbiota reshapes cancer immunotherapy efficacy: Mechanisms and therapeutic strategies. iMeta.

[4] Pearce E.L., Poffenberger M.C., Chang C.H., Jones R.G. (2013). Fueling immunity: insights into metabolism and lymphocyte function. Science.

[5] Buck M.D., Sowell R.T., Kaech S.M., Pearce E.L. (2017). Metabolic Instruction of Immunity. Cell.

[6] Pollizzi K.N., Powell J.D. (2014). Integrating canonical and metabolic signalling programmes in the regulation of T cell responses. Nat. Rev. Immunol..

[7] Belkaid Y., Harrison O.J. (2017). Homeostatic Immunity and the Microbiota. Immunity.

[8] Tran M., Huh J.R., Devlin A.S. (2025). The role of gut microbial metabolites in the T cell lifecycle. Nat. Immunol..

[9] Smith P.M., Howitt M.R., Panikov N., Michaud M., Gallini C.A., Bohlooly-Y M., Glickman J.N., Garrett W.S. (2013). The microbial metabolites, short-chain fatty acids, regulate colonic Treg cell homeostasis. Science.

[10] Luu M., Riester Z., Baldrich A., Reichardt N., Yuille S., Busetti A., Klein M., Wempe A., Leister H., Raifer H. (2021). Microbial short-chain fatty acids modulate CD8(+) T cell responses and improve adoptive immunotherapy for cancer. Nat. Commun..

[11] Rebeaud J., Vigne S., Bressoud V., Carre R., Ruiz F., Peter B., Phillips N.E., Siewert L., Roloff T., Egli A. (2025). Gut-derived metabolites drive Th17 cell pathogenicity in multiple sclerosis. Cell Rep..

[12] Clifford B.L., Sedgeman L.R., Williams K.J., Morand P., Cheng A., Jarrett K.E., Chan A.P., Brearley-Sholto M.C., Wahlström A., Ashby J.W. (2021). FXR activation protects against NAFLD via bile-acid-dependent reductions in lipid absorption. Cell Metab..

[13] Sorrentino G., Perino A., Yildiz E., El Alam G., Bou Sleiman M., Gioiello A., Pellicciari R., Schoonjans K. (2020). Bile Acids Signal via TGR5 to Activate Intestinal Stem Cells and Epithelial Regeneration. Gastroenterology.

[14] Petersen C., Satheesh Babu A.K., Della Lucia C.M., Paz H.A., Iglesias-Carres L., Zhong Y., Jalili T., Symons J.D., Shankar K., Neilson A.P. (2025). Gut microbes metabolize strawberry phytochemicals and mediate their beneficial effects on vascular inflammation. Gut Microbes.

[15] Klysz D.D., Fowler C., Malipatlolla M., Stuani L., Freitas K.A., Chen Y., Meier S., Daniel B., Sandor K., Xu P. (2024). Inosine induces stemness features in CAR-T cells and enhances potency. Cancer Cell.

[16] Elmarsafawi A.G., Hesterberg R.S., Fernandez M.R., Yang C., Darville L.N., Liu M., Koomen J.M., Phanstiel O., Atkins R., Mullinax J.E. (2023). Modulating the polyamine/hypusine axis controls generation of CD8+ tissue-resident memory T cells. JCI Insight.

[17] Sansbury S.E., Serebrenik Y.V., Lapidot T., Smith D.G., Burslem G.M., Shalem O. (2025). Pooled tagging and hydrophobic targeting of endogenous proteins for unbiased mapping of unfolded protein responses. Mol. Cell.

[18] Nishikawa K.K., Chen J., Acheson J.F., Harbaugh S.V., Huss P., Frenkel M., Novy N., Sieren H.R., Lodewyk E.C., Lee D.H. (2024). Highly multiplexed design of an allosteric transcription factor to sense new ligands. Nat. Commun..

[19] Li J., Ho D.J., Henault M., Yang C., Neri M., Ge R., Renner S., Mansur L., Lindeman A., Kelly B. (2022). DRUG-seq Provides Unbiased Biological Activity Readouts for Neuroscience Drug Discovery. ACS Chem. Biol..

[20] Ye C., Ho D.J., Neri M., Yang C., Kulkarni T., Randhawa R., Henault M., Mostacci N., Farmer P., Renner S. (2018). DRUG-seq for miniaturized high-throughput transcriptome profiling in drug discovery. Nat. Commun..

[21] Norton S., Gaspar J.M. (2025). DRAGoN: a robust pipeline for analyzing DRUG-seq datasets. Bioinform. Adv..

[22] Theodoris C.V., Xiao L., Chopra A., Chaffin M.D., Al Sayed Z.R., Hill M.C., Mantineo H., Brydon E.M., Zeng Z., Liu X.S., Ellinor P.T. (2023). Transfer learning enables predictions in network biology. Nature.

[23] Ahlmann-Eltze C., Huber W., Anders S. (2025). Deep-learning-based gene perturbation effect prediction does not yet outperform simple linear baselines. Nat. Methods.

[24] DeMeo B., Nesbitt C., Miller S.A., Burkhardt D.B., Lipchina I., Fu D., Holderrieth P., Kim D., Kolchenko S., Szalata A. (2025). Active learning framework leveraging transcriptomics identifies modulators of disease phenotypes. Science.

[25] Ma S., Ming Y., Wu J., Cui G. (2024). Cellular metabolism regulates the differentiation and function of T-cell subsets. Cell. Mol. Immunol..

[26] Longo J., Watson M.J., Williams K.S., Sheldon R.D., Jones R.G. (2025). Nutrient allocation fuels T cell-mediated immunity. Cell Metab..

[27] Kaminskiy Y., Melenhorst J.J. (2022). STAT3 Role in T-Cell Memory Formation. Int. J. Mol. Sci..

[28] Villarino A.V. (2023). Transcriptional programing of T cell metabolism by STAT family transcription factors. Eur. J. Immunol..

[29] Kishton R.J., Sukumar M., Restifo N.P. (2017). Metabolic Regulation of T Cell Longevity and Function in Tumor Immunotherapy. Cell Metab..

[30] Xu T., Stewart K.M., Wang X., Liu K., Xie M., Ryu J.K., Li K., Ma T., Wang H., Ni L. (2017). Metabolic control of T(H)17 and induced T(reg) cell balance by an epigenetic mechanism. Nature.

[31] Lotfollahi M., Wolf F.A., Theis F.J. (2019). scGen predicts single-cell perturbation responses. Nat. Methods.

[32] Ding S., Li J., Luo R., Cui H., Wang B., Chen R. (2026). scGPT: end-to-end protocol for fine-tuned retinal cell type annotation. Nat. Protoc..

[33] Cui H., Wang C., Maan H., Pang K., Luo F., Duan N., Wang B. (2024). scGPT: toward building a foundation model for single-cell multi-omics using generative AI. Nat. Methods.

[34] Jutel M., Watanabe T., Klunker S., Akdis M., Thomet O.A., Malolepszy J., Zak-Nejmark T., Koga R., Kobayashi T., Blaser K., Akdis C.A. (2001). Histamine regulates T-cell and antibody responses by differential expression of H1 and H2 receptors. Nature.

[35] Tannahill G.M., Curtis A.M., Adamik J., Palsson-McDermott E.M., McGettrick A.F., Goel G., Frezza C., Bernard N.J., Kelly B., Foley N.H. (2013). Succinate is an inflammatory signal that induces IL-1beta through HIF-1alpha. Nature.

[36] Atarashi K., Nishimura J., Shima T., Umesaki Y., Yamamoto M., Onoue M., Yagita H., Ishii N., Evans R., Honda K., Takeda K. (2008). ATP drives lamina propria T(H)17 cell differentiation. Nature.

[37] Hayashi T., Mo J.H., Gong X., Rossetto C., Jang A., Beck L., Elliott G.I., Kufareva I., Abagyan R., Broide D.H. (2007). 3-Hydroxyanthranilic acid inhibits PDK1 activation and suppresses experimental asthma by inducing T cell apoptosis. Proc. Natl. Acad. Sci. USA.

[38] Zelante T., Iannitti R.G., Cunha C., De Luca A., Giovannini G., Pieraccini G., Zecchi R., D'Angelo C., Massi-Benedetti C., Fallarino F. (2013). Tryptophan catabolites from microbiota engage aryl hydrocarbon receptor and balance mucosal reactivity via interleukin-22. Immunity.

[39] Calabrese E.J. (2018). Hormesis: Path and Progression to Significance. Int. J. Mol. Sci..

[40] Rothhammer V., Quintana F.J. (2019). The aryl hydrocarbon receptor: an environmental sensor integrating immune responses in health and disease. Nat. Rev. Immunol..

[41] Ducker G.S., Rabinowitz J.D. (2017). One-Carbon Metabolism in Health and Disease. Cell Metab..

[42] Xavier R.J., Podolsky D.K. (2007). Unravelling the pathogenesis of inflammatory bowel disease. Nature.

[43] Xu Y., Song D., Su Y., Chen J., Wu L., Lian H., Hai N., Li J., Jiang J., Zhao J. (2023). Pharmacology-based molecular docking of 4-methylcatechol and its role in RANKL-mediated ROS/Keap1/Nrf2 signalling axis and osteoclastogenesis. Biomed. Pharmacother..

[44] Ng M.Y., Hagen T. (2025). A strategy for liver selective NRF2 induction via cytochrome P450-activated prodrugs with low activity in hypoxia. J. Biol. Chem..

[45] Zheng L.T., Ryu G.M., Kwon B.M., Lee W.H., Suk K. (2008). Anti-inflammatory effects of catechols in lipopolysaccharide-stimulated microglia cells: inhibition of microglial neurotoxicity. Eur. J. Pharmacol..

[46] Aoki R., Aoki-Yoshida A., Suzuki C., Takayama Y. (2018). Indole-3-Pyruvic Acid, an Aryl Hydrocarbon Receptor Activator, Suppresses Experimental Colitis in Mice. J. Immunol..

[47] Pernomian L., Duarte-Silva M., de Barros Cardoso C.R. (2020). The Aryl Hydrocarbon Receptor (AHR) as a Potential Target for the Control of Intestinal Inflammation: Insights from an Immune and Bacteria Sensor Receptor. Clin. Rev. Allergy Immunol..

[48] Kamata K., Hara A., Minaga K., Yoshikawa T., Kurimoto M., Sekai I., Okai N., Omaru N., Masuta Y., Otsuka Y. (2023). Activation of the aryl hydrocarbon receptor inhibits the development of experimental autoimmune pancreatitis through IL-22-mediated signaling pathways. Clin. Exp. Immunol..

[49] Zhao H., Guo P., Zuo Y., Wang Y., Zhao H., Lan T., Xue M., Zhang H., Liang H. (2022). Folic acid intervention changes liver Foxp3 methylation and ameliorates the damage caused by Th17/Treg imbalance after long-term alcohol exposure. Food Funct..

[50] Shang M., Yang H., Yang R., Chen T., Fu Y., Li Y., Fang X., Zhang K., Zhang J., Li H. (2021). The folate cycle enzyme MTHFD2 induces cancer immune evasion through PD-L1 up-regulation. Nat. Commun..

[51] Courtemanche C., Elson-Schwab I., Mashiyama S.T., Kerry N., Ames B.N. (2004). Folate deficiency inhibits the proliferation of primary human CD8+ T lymphocytes in vitro. J. Immunol..

[52] Yamaguchi T., Hirota K., Nagahama K., Ohkawa K., Takahashi T., Nomura T., Sakaguchi S. (2007). Control of immune responses by antigen-specific regulatory T cells expressing the folate receptor. Immunity.

[53] Mirji G., Bhat S.A., El Sayed M., Reiser S.K., Gavara S.P., Ye Y., Miyamoto T., Zhang W., Vogel P., Cassel J. (2026). Aromatic microbial metabolite hippuric acid enhances inflammatory responses in macrophages via TLR-MyD88 signaling and lipid remodeling. Cell Rep..

[54] Neurath M.F. (2014). Cytokines in inflammatory bowel disease. Nat. Rev. Immunol..

[55] Li H.B., Tong J., Zhu S., Batista P.J., Duffy E.E., Zhao J., Bailis W., Cao G., Kroehling L., Chen Y. (2017). m(6)A mRNA methylation controls T cell homeostasis by targeting the IL-7/STAT5/SOCS pathways. Nature.

[56] Smith T., Heger A., Sudbery I. (2017). UMI-tools: modeling sequencing errors in Unique Molecular Identifiers to improve quantification accuracy. Genome Res..

[57] Chen S., Zhou Y., Chen Y., Gu J. (2018). fastp: an ultra-fast all-in-one FASTQ preprocessor. Bioinformatics.

[58] Dobin A., Davis C.A., Schlesinger F., Drenkow J., Zaleski C., Jha S., Batut P., Chaisson M., Gingeras T.R. (2013). STAR: ultrafast universal RNA-seq aligner. Bioinformatics.

[59] Liao Y., Smyth G.K., Shi W. (2014). featureCounts: an efficient general purpose program for assigning sequence reads to genomic features. Bioinformatics.

[60] Li H., Handsaker B., Wysoker A., Fennell T., Ruan J., Homer N., Marth G., Abecasis G., Durbin R., 1000 Genome Project Data Processing Subgroup (2009). The Sequence Alignment/Map format and SAMtools. Bioinformatics.

[61] Hao Y., Stuart T., Kowalski M.H., Choudhary S., Hoffman P., Hartman A., Srivastava A., Molla G., Madad S., Fernandez-Granda C., Satija R. (2024). Dictionary learning for integrative, multimodal and scalable single-cell analysis. Nat. Biotechnol..

[62] Love M.I., Huber W., Anders S. (2014). Moderated estimation of fold change and dispersion for RNA-seq data with DESeq2. Genome Biol..

[63] Xu S., Hu E., Cai Y., Xie Z., Luo X., Zhan L., Tang W., Wang Q., Liu B., Wang R. (2024). Using clusterProfiler to characterize multiomics data. Nat. Protoc..

[64] Aibar S., González-Blas C.B., Moerman T., Huynh-Thu V.A., Imrichova H., Hulselmans G., Rambow F., Marine J.C., Geurts P., Aerts J. (2017). SCENIC: single-cell regulatory network inference and clustering. Nat. Methods.

[65] Hänzelmann S., Castelo R., Guinney J. (2013). GSVA: gene set variation analysis for microarray and RNA-seq data. BMC Bioinf..

[66] Qiu X., Mao Q., Tang Y., Wang L., Chawla R., Pliner H.A., Trapnell C. (2017). Reversed graph embedding resolves complex single-cell trajectories. Nat. Methods.

[67] Kang M., Armenteros J.J.A., Gulati G.S., Gleyzer R., Avagyan S., Brown E.L., Zhang W., Usmani A., Earland N., Wu Z. (2024). Mapping single-cell developmental potential in health and disease with interpretable deep learning. bioRxiv.

[68] Miao Y.R., Xia M., Luo M., Luo T., Yang M., Guo A.Y. (2022). ImmuCellAI-mouse: a tool for comprehensive prediction of mouse immune cell abundance and immune microenvironment depiction. Bioinformatics.

[69] Zhao Y., Ma Y., Li H., Chen M., Yang S., Xu Y., Zhang Q., Jiao B., Tan Y. (2025). An atlas of transcriptomic changes in human immune cells driven by 364 endogenous and gut-microbiota-derived metabolites. Sci. Rep..

[70] Senger D.R., Li D., Jaminet S.C., Cao S. (2016). Activation of the Nrf2 Cell Defense Pathway by Ancient Foods: Disease Prevention by Important Molecules and Microbes Lost from the Modern Western Diet. PLoS One.

[71] Chen Q.Q., Liu K., Shi N., Ma G., Wang P., Xie H.M., Jin S.J., Wei T.T., Yu X.Y., Wang Y. (2023). Neuraminidase 1 promotes renal fibrosis development in male mice. Nat. Commun..

[72] Lapin I.P., Politi V. (1993). Anxiolytic effect of indole-3-pyruvic acid (IPA) in mice. Pharmacol. Res..

[73] Wirtz S., Neufert C., Weigmann B., Neurath M.F. (2007). Chemically induced mouse models of intestinal inflammation. Nat. Protoc..

[74] Danecek P., Bonfield J.K., Liddle J., Marshall J., Ohan V., Pollard M.O., Whitwham A., Keane T., McCarthy S.A., Davies R.M., Li H. (2021). Twelve years of SAMtools and BCFtools. GigaScience.

