# Supplementary material for: A systematic transcriptomic atlas and predictive framework for metabolite-driven T cell state transitions
Source: iScience. 2026 Sep 18;29(10):117527. doi: 10.1016/j.isci.2026.117527 (PMC13626933; doi:10.1016/j.isci.2026.117527)
Supplement: Supplementary file 1 [file mmc1.pdf]

iScience, Volume *29*

Supplemental information

A systematic transcriptomic atlas and predictive framework for metabolite-driven T cell state transitions

Yiyang Xu, Yiheng Zhao, Sizhe Yang, Huimin Li, Xiangrong Diao, Qianqian Zhang, Shuting Yu, Shuai Wang, Fangying Jiang, Qiang Wang, Yuhua Ma, Bo Jiao, Yun Tan, and Yabin Liu

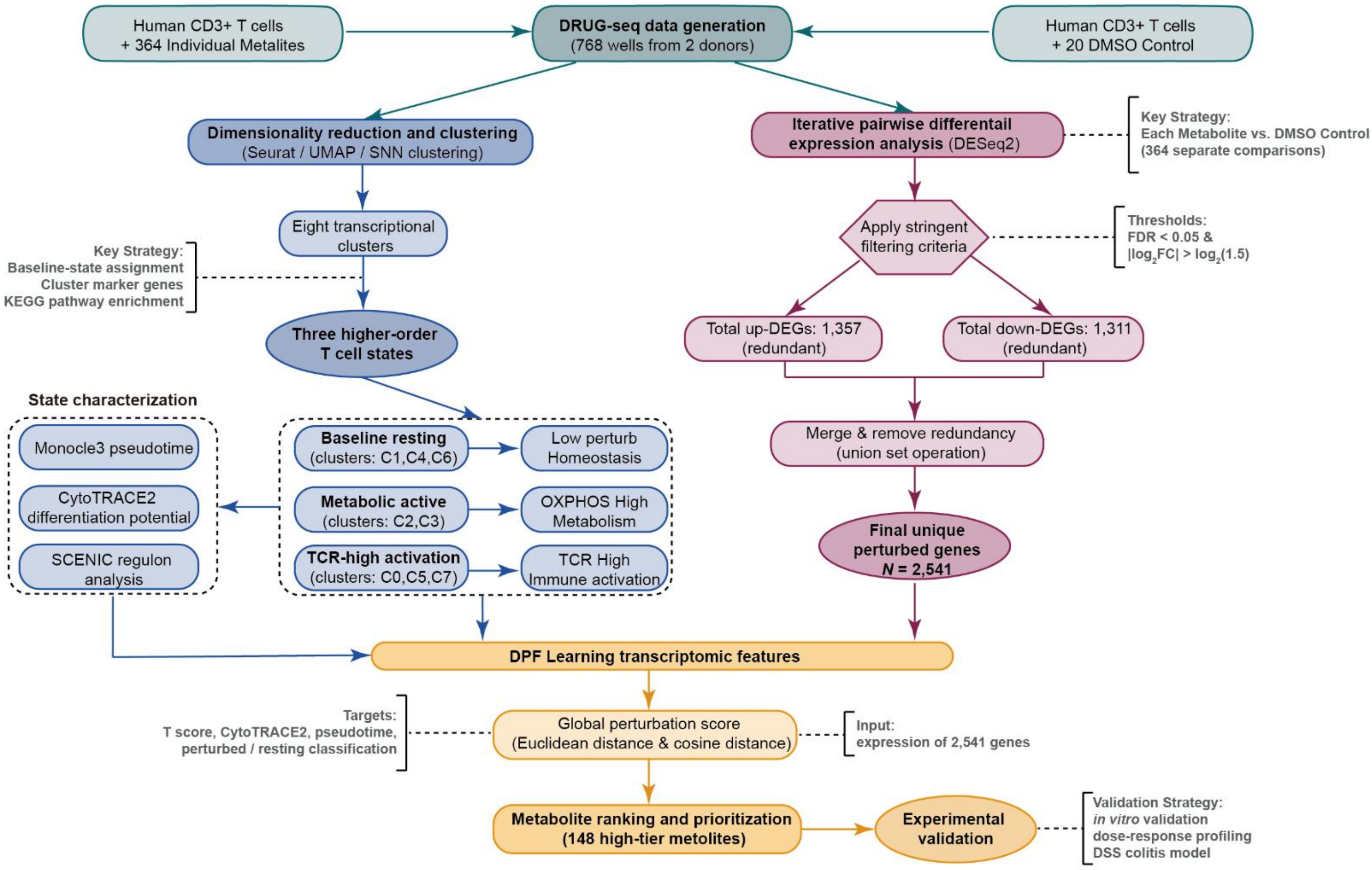

Figure S1. Integrated analytical framework for defining metabolite-responsive T cell states and DPF-based metabolite prioritization.

Schematic overview of the study workflow. Human CD3$^+$ T cells from two independent donors were treated with 364 individual metabolites or matched DMSO controls, generating 768 DRUG-seq profiles. Dimensionality reduction and clustering identified eight transcriptional clusters, which were grouped into three higher-order T cell states—basal resting, metabolically active, and TCR-highly activated—based on baseline-state assignment, cluster marker genes, and KEGG pathway enrichment. State-associated features were further characterized using Monocle3 pseudotime, CytoTRACE2 differentiation potential, and SCENIC regulon analysis. In parallel, pairwise differential expression analysis comparing each metabolite with matched DMSO controls was used to construct a nonredundant Perturbed Gene Universe comprising 2,541 genes. These gene-expression profiles and state-related objectives were incorporated into DPF multi-task learning to generate latent perturbation representations and global perturbation scores, enabling ranking of all screened metabolites and prioritization of 148 high-tier candidates for in vitro validation, concentration profiling, and testing in the DSS colitis model.

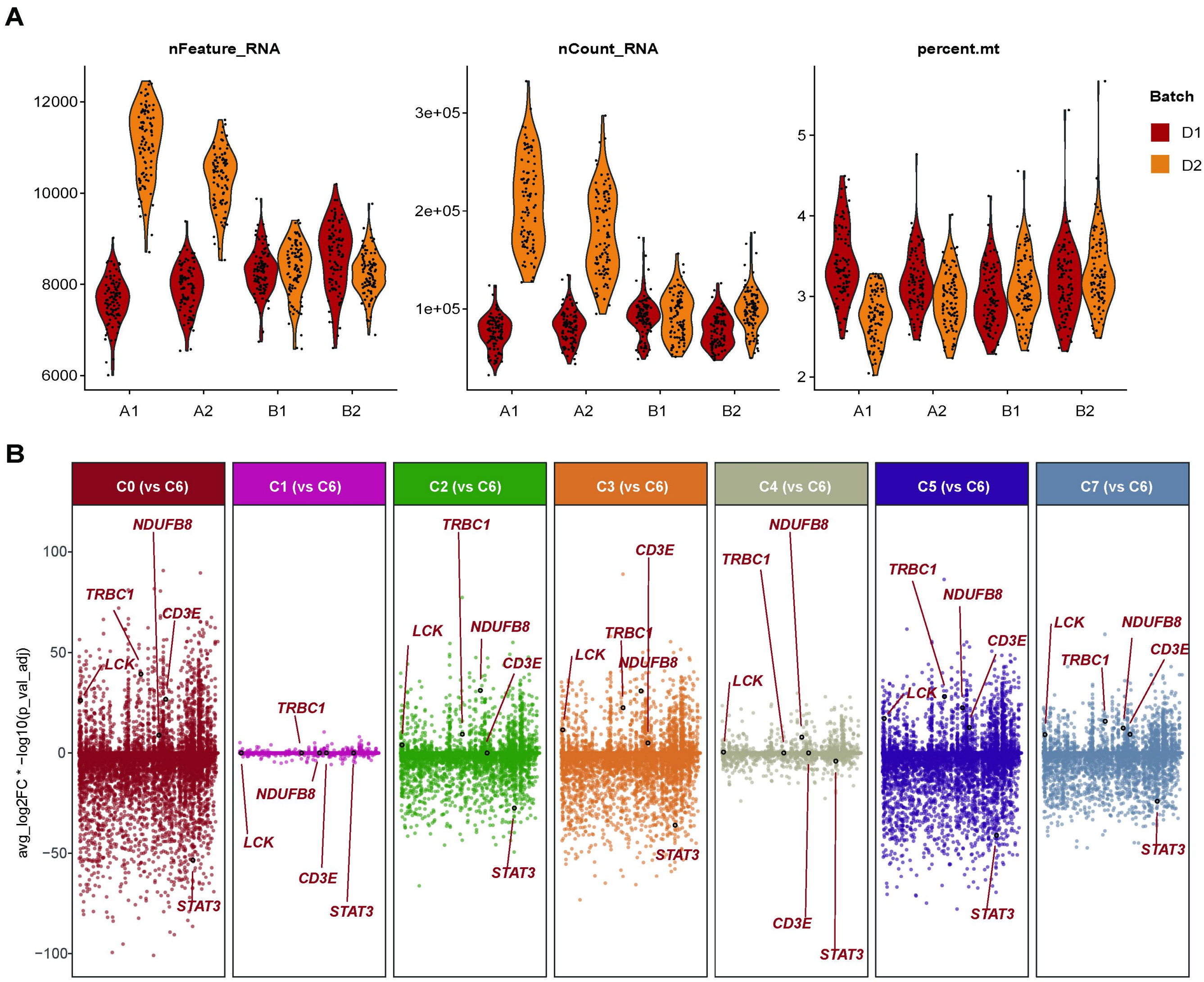

Figure S2. Quality control assessment and cluster-specific differential expression analysis.

(A) Quality control metrics for the DRUG-seq screen, including the number of detected features (nFeature_RNA), UMI counts (nCount_RNA), and mitochondrial gene percentage (percent.mt), across the four DRUG-seq plates (A1, A2, B1, B2). Each dot represents a single well (one perturbation sample). Colors indicate the two experimental batches (D1, red; D2, orange). Variations in library size and sequencing depth across plates (for example, between A1 and A2) indicate technical inter-plate variation and the need for normalization. These batch effects were subsequently mitigated using the SCTransform (SCT) integration workflow in downstream analyses. **(B)** Differential expression scatter plots for each cluster (C0-C5, C7) compared with the homeostatic baseline cluster C6. Adjusted *P* values were derived from Seurat FindMarkers using the Wilcoxon rank-sum test with Bonferroni correction. Representative genes are labeled, including *STAT3, TRBC1, LCK, CD3E,* and *NDUFB8*.

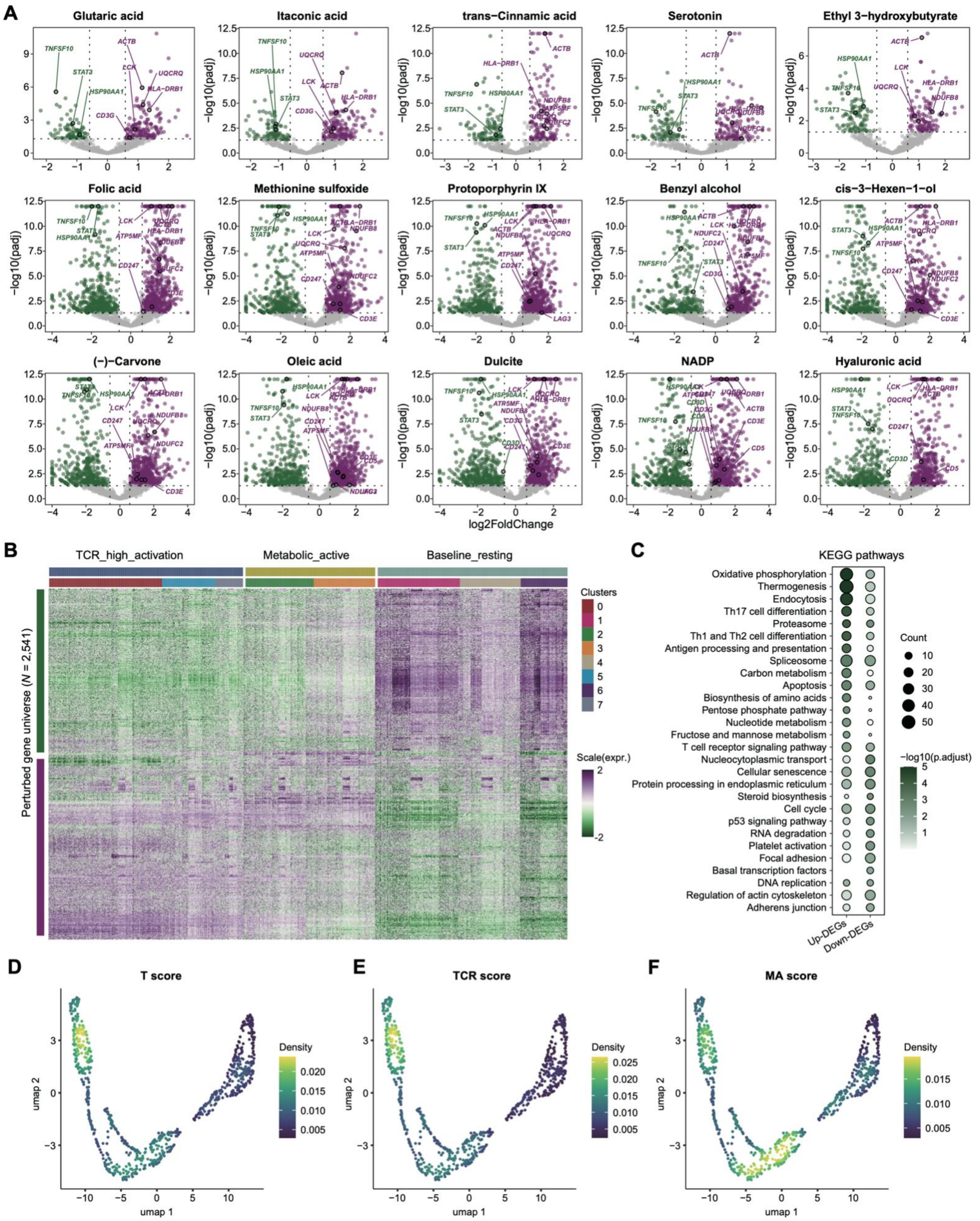

Figure S3. Metabolite-induced transcriptional responses and differential expression profiles.

(A) Volcano plots showing the differential gene expression profiles for the representative top-ranked metabolites. Purple dots indicate significantly upregulated genes, and green dots indicate downregulated genes compared to DMSO controls, with key immune and metabolic markers labeled. *P* values were calculated using the DESeq2 Wald test and adjusted for multiple comparisons using the BH method. **(B)**

Heatmap of the “perturbed gene universe” (2,541 genes that are differentially expressed in at least one metabolite condition versus DMSO), showing scaled expression across all compound treatments. **(C)** KEGG pathway enrichment analysis performed separately on the upregulated and downregulated gene sets derived from the perturbed gene universe, revealing distinct activation and metabolic pathways. *P* values were calculated using a hypergeometric test and adjusted for multiple comparisons using the BH method. **(D–F)** UMAP projections of all metabolite conditions colored by functional scores derived from the perturbed gene signatures: T cell activation score (D), TCR signaling score (E), and Metabolic activation score (F). Colors reflect the density/strength of each score in the low-dimensional space.

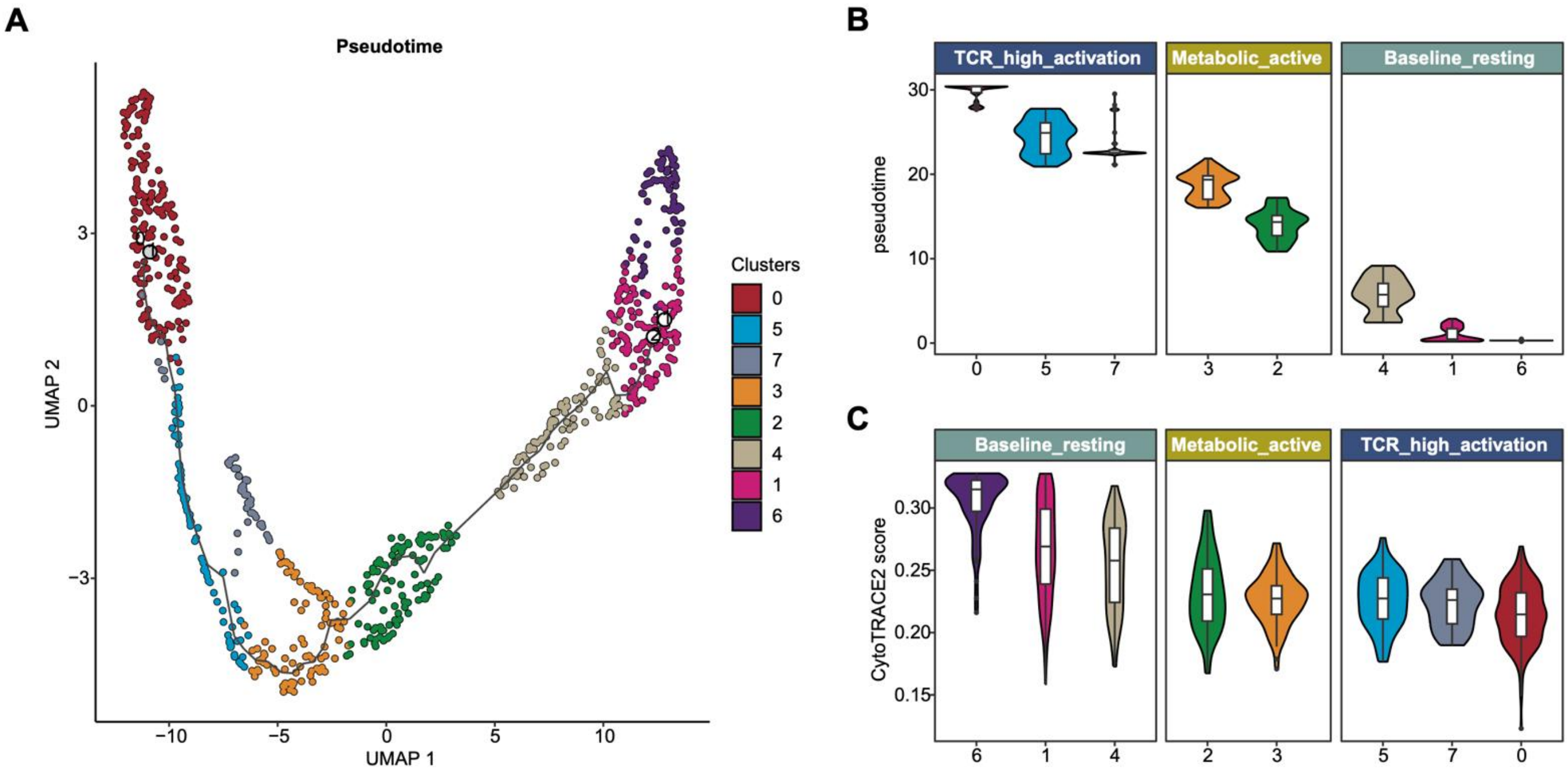

Figure S4. Trajectory inference and differentiation potential analysis of metabolite-induced T cell states.

(A) Monocle3 differentiation trajectory inference visualizing the developmental route of T cells. Cells are colored by their transcriptional cluster identities (C0–C7), illustrating the topological arrangement of distinct cell states along the trajectory. **(B)** Violin plots displaying the distribution of Monocle3-inferred pseudotime values across the three functional states (Baseline_resting, Metabolic_active, TCR_high_activation) and their constituent clusters. Higher pseudotime values correspond to the progression towards terminal differentiation. **(C)** Violin plots showing the distribution of CytoTRACE2 scores (predicting developmental potential/stemness) across the defined functional states and clusters. The shift in scores corroborates the hierarchical differentiation from the resting state to the activated effector states. The width of the violin plots represent the kernel density estimation of the data distribution. Within each violin, the box extends from the 25th percentile (Q1) to the 75th percentile (Q3), with the central line indicating the median. The whiskers extend to the minimum and maximum values within 1.5 × IQR from the lower and upper quartiles, respectively.

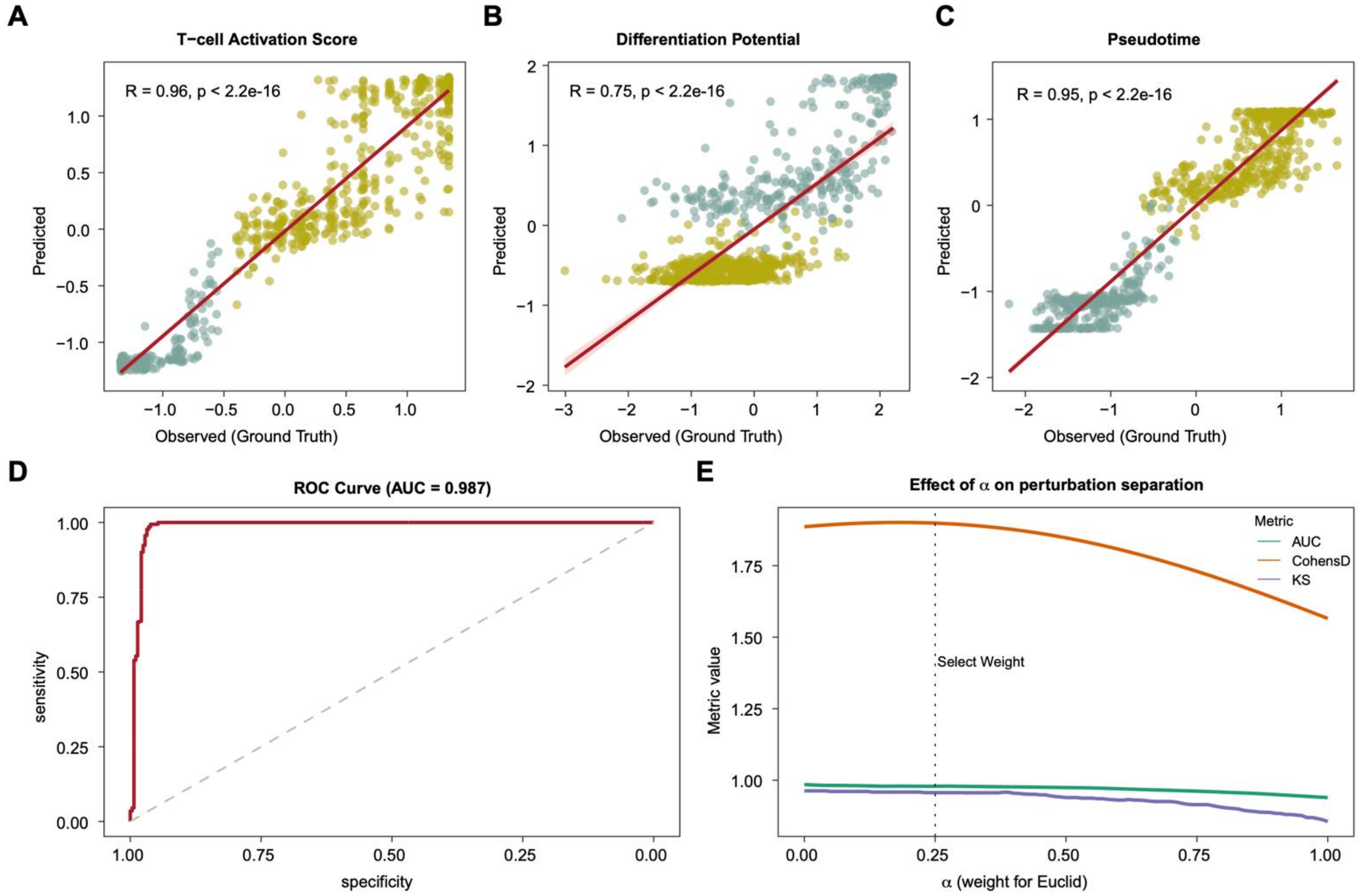

Figure S5. Performance evaluation of the DRUG-seq PerturbFormer (DPF) model.

(A-C) Scatter plots showing the Pearson correlation between ground-truth (observed) and DPF-predicted values for three continuous phenotypic scores: T-cell activation score (A), differentiation potential inferred from CytoTRACE2 (B), and pseudotime inferred with Monocle3 (C). Pearson's r and two-sided P value are shown. **(D)** Receiver operating characteristic (ROC) curve showing the classification performance of DPF (AUC = 0.987, 95% CI 0.976–0.998). **(E)** Parameter sensitivity analysis showing the impact of the weighting parameter α on the model's ability to separate perturbed from resting states, assessed by AUC, Cohen's D, and KS statistics.

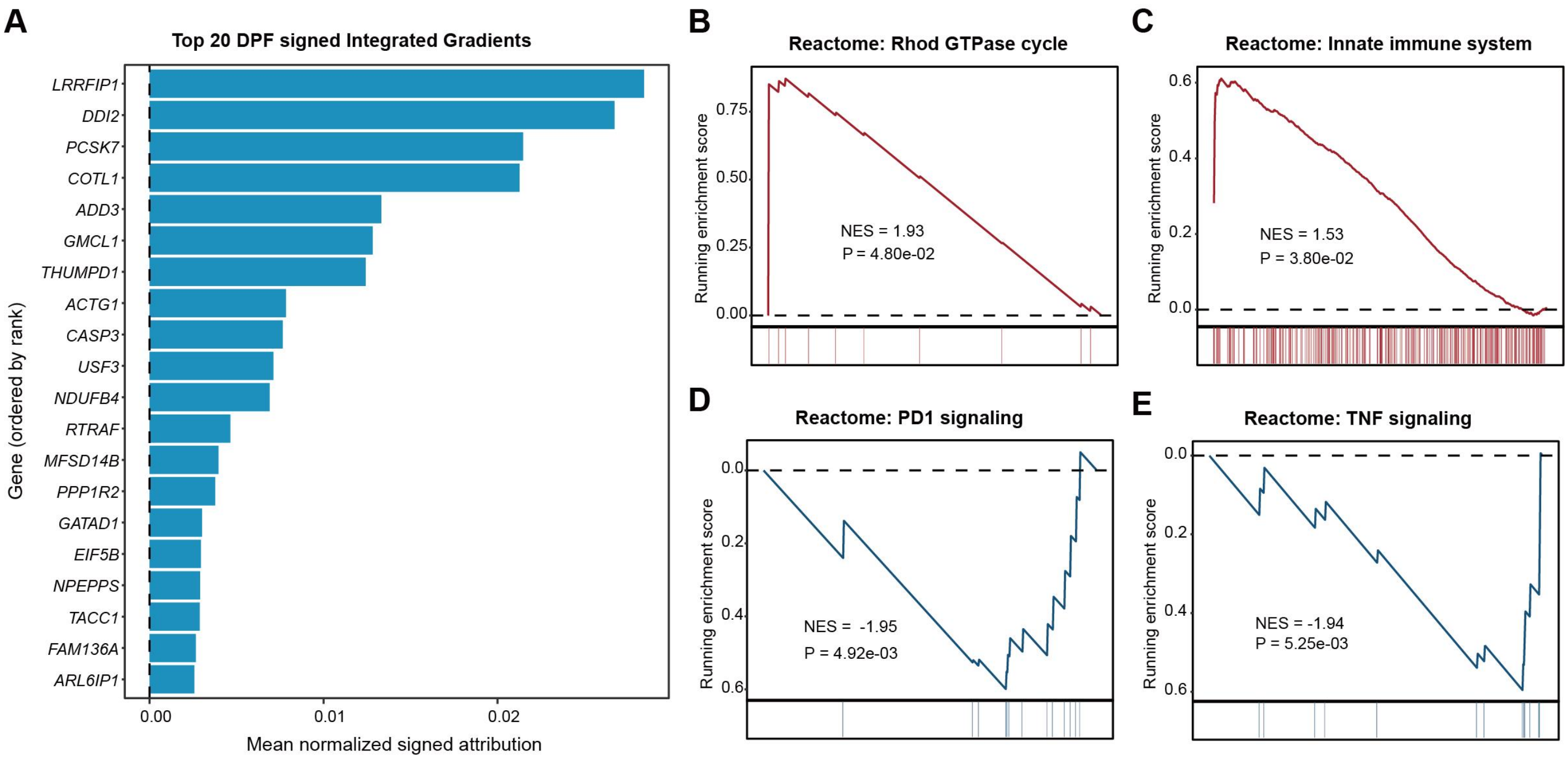

Figure S6. Gene- and pathway-level attribution of the DPF-derived perturbation score.

(A) Top 20 gene attributions ranked by the mean signed Integrated Gradient value across analyzed samples. Values indicate contributions toward a higher model-derived perturbation score. **(B–E)** Running enrichment plots generated from the signed gene-attribution ranking for the Reactome Rho GTPase cycle (B), innate immune system (C), PD-1 signaling (D), and TNF signaling (E) gene sets. Vertical ticks indicate the positions of genes belonging to each pathway within the ranked attribution list. Normalized enrichment scores (NES) and nominal *P* values are shown. These enrichment results represent exploratory patterns in model attribution and should not be interpreted as expression-based evidence of pathway activation or inhibition.

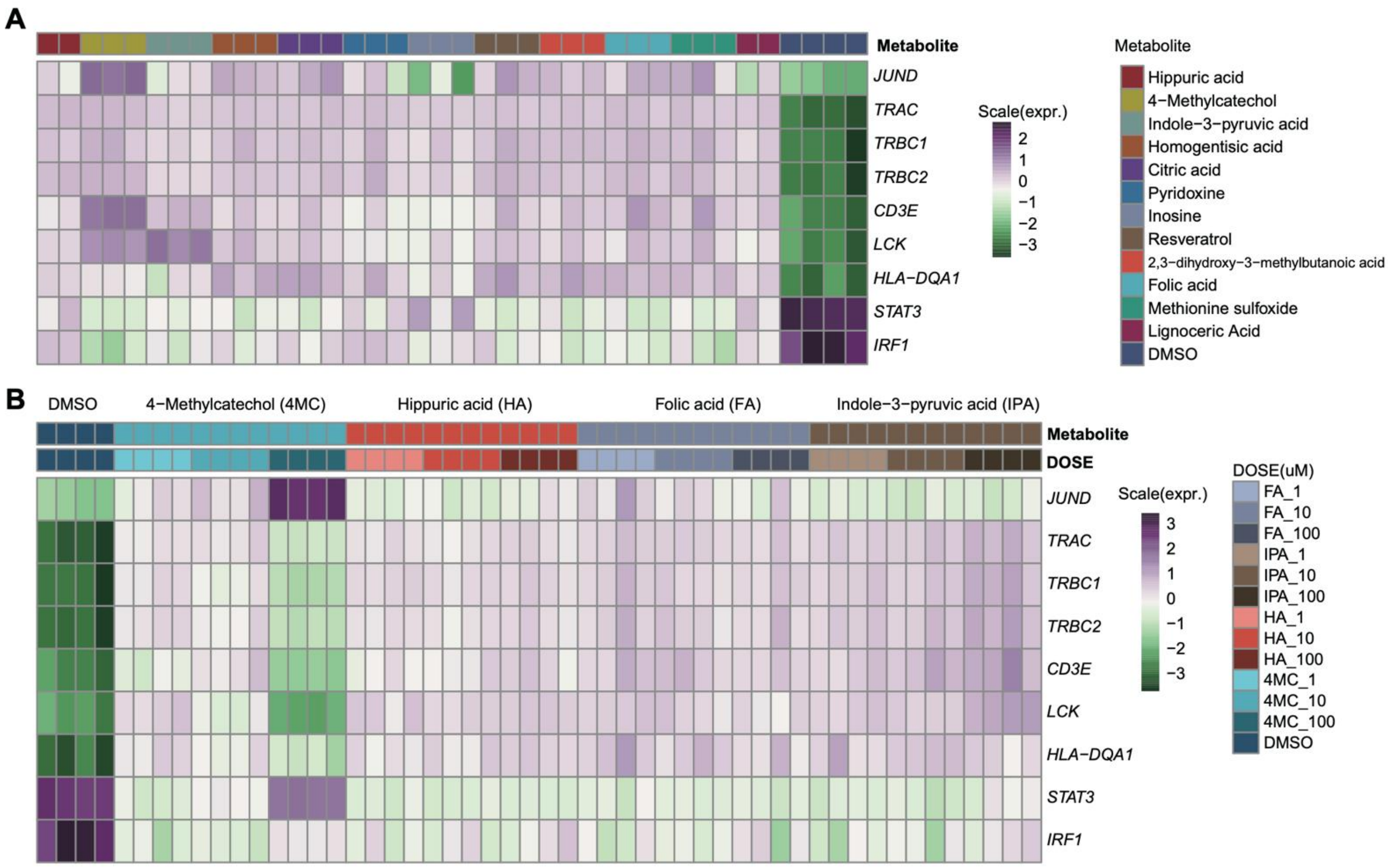

Figure S7. Transcriptional validation of key metabolite hits and dose-dependent responses.

(A) Heatmap displaying the expression profiles of representative T cell regulatory genes (e.g., *JUND, TRAC, CD3E, LCK, STAT3*) across 12 representative metabolites identified from the screen compared to DMSO. **(B)** Dose-response heatmap for the four selected candidate metabolites: Folic acid (FA), Indole-3-pyruvic acid (IPA), Hippuric acid (HA), and 4-Methylcatechol (4MC). The plot shows the expression changes of key marker genes across three increasing concentrations (1, 10, and 100 µM) relative to DMSO, confirming the dose-dependent transcriptional modulation of these compounds.

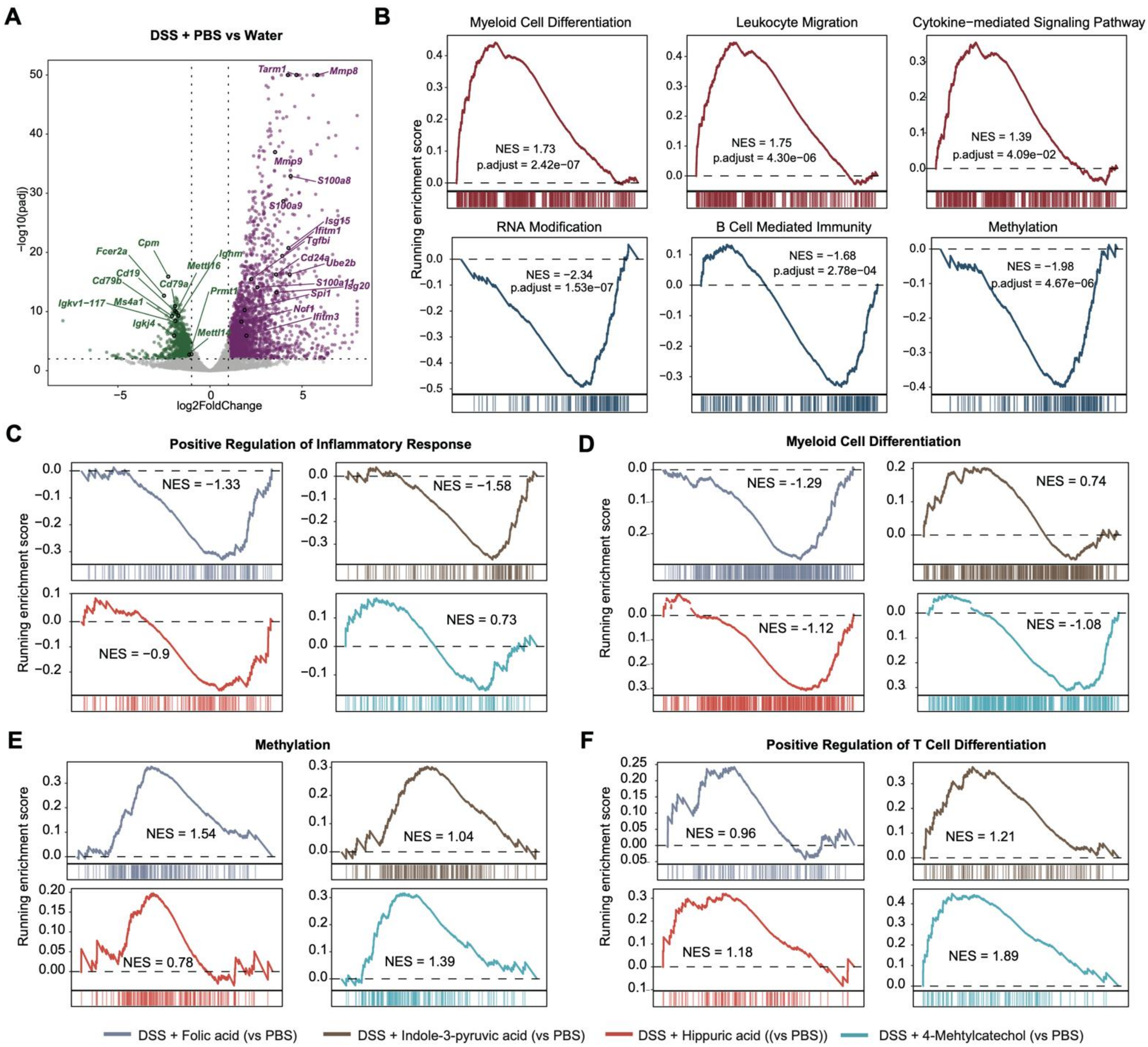

Figure S8. Metabolite treatment attenuates DSS-induced colitis–associated transcriptional changes.

(A) Volcano plot comparing gene expression between DSS-induced colitis (DSS + PBS) and water-treated controls. The x-axis shows $\log_2$ fold change (DSS + PBS vs Water) and the y-axis shows $-\log_{10}$(adjusted p value). Classical inflammatory markers (e.g., *S100a8*, *S100a9*) are strongly upregulated, whereas B-cell–associated genes (e.g., *Cd19*, *Cd79a*) are downregulated, confirming successful induction of colitis at the transcriptomic level. **(B)** GSEA normalized enrichment scores (NES) for representative pathways in DSS + PBS vs Water. DSS treatment positively enriches inflammatory and myeloid programs (Myeloid cell differentiation, Leukocyte migration, Cytokine-mediated signaling pathway) and negatively enriches homeostatic/lymphoid or metabolic programs (RNA modification, B cell mediated immunity, Methylation). **(C–D)** GSEA of inflammatory pathways in metabolite-treated DSS mice versus DSS controls. (C) “Positive regulation of inflammatory response” and (D) “Myeloid cell differentiation” generally show negative NES after treatment, indicating suppression of innate inflammatory programs, with compound-specific differences in the degree of inhibition. **(E–F)** GSEA of homeostatic and adaptive immune pathways in

metabolite-treated DSS mice versus DSS controls. (E) “Methylation” and (F) “Positive regulation of T cell differentiation” show positive NES across effective treatments (e.g., folic acid and 4-methylcatechol), suggesting restoration of T-cell homeostasis and epigenetic regulatory programs disrupted in DSS colitis.
